# Suspended thin-film lithium niobate modulator for broadband mid-infrared light modulation and frequency comb generation

**DOI:** 10.1038/s41467-026-77674-y

**Published:** 2026-09-17

**Authors:** Chun-Ho Lee, Xinyi Ren, Xinzhou Su, Wonho Lee, Zile Jiang, Yue Yu, Huibin Zhou, Yue Zuo, Shaoyuan Ou, Reshma Kopparapu, Adam T. Heiniger, Moshe Tur, Alan E. Willner, Zaijun Chen, Mengjie Yu

**Affiliations:** 1 Department of Electrical Engineering and Computer Sciences, University of California, Berkeley, CA USA; 2 Ming Hsieh Department of Electrical and Computer Engineering, University of Southern California, Los Angeles, CA USA; 3 PHY Research Lab, Intel lab, Hillsboro, OR USA; 4 Materials Sciences Division, Lawrence Berkeley National Laboratory, Berkeley, California USA; 5 TOPTICA Photonics Inc., Pittsford, NY USA; 6 School of Electrical Engineering, Tel Aviv University, Ramat Aviv, Israel; 7 Faculty of Electrical Engineering, Holon Institute of Technology, Holon, Israel

**Keywords:** Mid-infrared photonics, Nanophotonics and plasmonics, Nonlinear optics

## Abstract

The mid-infrared (MIR) spectral regime is central to applications including remote sensing, precision spectroscopy, higher harmonic generation, and free-space optical communication. However, coherent and broadband MIR modulation remains challenging owing to high optical loss, limited bandwidth, and large drive voltages in existing platforms. Here, we overcome the challenges by deploying a suspended thin-film lithium-niobate (TFLN) based electro-optic (EO) platform co-designed with high-performance traveling-wave microwave (MW) electrodes. We demonstrate a record-low *V*_*π*,*DC*_ of 2.3 to 4.3 V over a broadband MIR bandwidth from 2.4 to 3.6 μm, and a 2.3-dB EO bandwidth of 40 GHz (extracted 3-dB bandwidth of 52 GHz), yielding a figure-of-merit of 19.3 GHz/V–more than an order of magnitude higher than the state-of-the-art. We demonstrate, for the first time, high-frequency *V*_*π*,*MW*_ of 4.5-6.5 V in the 27-34 GHz range, and frequency-agile MIR EO frequency comb generation with a 10-dB optical bandwidth over 0.8 THz using a suspended phase modulator of 4-cm active modulation length. We further validate the platform in a free-space optical communication link. Our results establish a monolithic MIR photonic platform capable of powerful EO modulation and spectral synthesis, and present a significant step towards reconfigurable MIR sensing and communication systems on chip.

## Abstract (web summary)

Mid-infrared electro-optic modulation is crucial for sensing, spectroscopy, nonlinear optics, and free-space communication. Here, authors demonstrate a suspended thin-film lithium-niobate platform operating from 2.4-3.6 μm with 2.3–4.3 V *V*_*π*_, 50 GHz bandwidth, and frequency-agile comb generation

## Introduction

The rapidly growing amount of data being transmitted across networks is creating a significant demand for new wireless communication technologies to support higher data rates, lower latency, higher connection density, and global coverage^1^. Free space optical communication is an emerging technology for data transfer to remote assets through atmospheric communication channels, addressing the broadband connectivity bottlenecks for space and terrestrial applications^2,3^ while offering tremendous advantages over radio-frequency carriers due to its high bandwidth, immunity to electromagnetic interferences, low latency, and multiplexing features.

The MIR spectral range from 3 to 5 μm and long-wave infrared (LIR) from 8 to 12 μm are actively being investigated as free space optical carriers since it has the lower absorption in the atmosphere, the higher tolerance to adverse weather conditions (such as dust, haze, and low-altitude clouds), and less phase-front distortion by turbulence effects compared to the near-infrared (NIR), mm-wave, and THz-waves^4–14^. There are recent developments of the optoelectronic devices based on subsequent intersubband transitions and stark effect at LIR, such as quantum cascade laser (QCL), quantum-well infrared detector (QWIP) and quantum cascade detector, however the efficiency of unipolar device is significantly worse in the MIR region^15–19^. Existing approaches to modulate the MIR light include direct current modulation of a QCL at a limited bandwidth of a few GHz at room temperature^16,20–24^, and nonlinear parametric conversion of the near-infrared light which suffers from limited optical conversion bandwidth and efficiency as well as requires external power-hungry pump lasers^25^. Alternatively, EO material could be used to modulate the refractive index or optical absorption via external voltages. The MIR EO modulators have been demonstrated in silicon^26^, silicon on lithium niobate (LN)^8,27^, titanium dioxide on LN^28^, ion diffused waveguide on LN^29^, black phosphorous on silicon^30,31^, germanium on silicon^32,33^, and barium titanate^34^. However, none of the material platforms offers beyond a few GHz modulation speed and CMOS-compatible half-wave voltages. In addition, traditional silicon-based modulators have inevitable high optical losses due to the free carrier absorption at longer optical wavelengths. Therefore, a compact, high-speed and efficient EO modulator in the MIR is still missing.

**Fig. 1:**
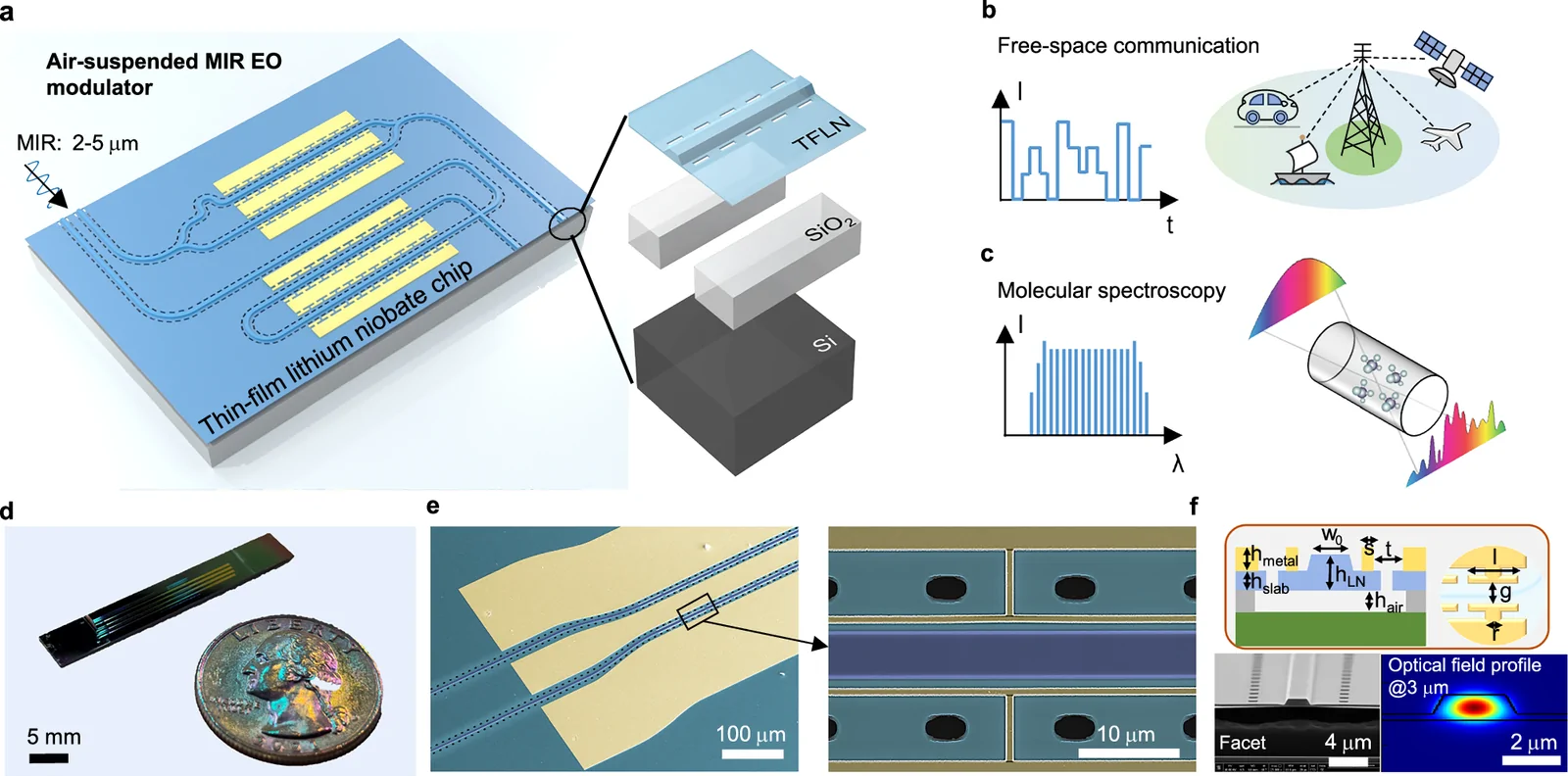
Monolithic mid-infrared (MIR) optoelectronic platform on TFLN. **a** Air-suspended MIR traveling-wave-based amplitude and phase electro-optic (EO) modulators on the TFLN. The inset shows the cross-sectional view of the optical layer, which consists of a ridge TFLN waveguide sitting on an oxide-on-Si substrate. The oxide layer is removed underneath the waveguide area through air holes on the TFLN slab layer etched via a second etching process. High-performance optoelectronic interface in the MIR would enable **b** high-bandwidth data links via free space communication and **c** frequency-agile EO frequency combs for molecular spectroscopy. **d** A photographic image of the fabricated MIR modulator chip on 800-nm X-cut TFLN. **e** A scanning-electron-microscopy (SEM) image of suspended photonic waveguides and coplanar microwave transmission line. Segmented slow-wave electrode design (zoom in, right) is applied to reduce the microwave loss and achieve impedance matching and velocity matching with the optical field while being compatible with a low optical propagation loss. Air hole arrays are optimized and placed between the metal segments to release the adjacent photonic waveguides. **f** Crosssection of the suspended device where the metal gap (g), waveguide height (*h*_*LN*_), slab thickness (*h*_*slab*_), metal thickness (*h*_*metal*_), waveguide width (*w*_0_), the air gap under the waveguide (*h*_*air*_) and the segmented electrode parameters (s, t, r, l) are 6.5, 0.8, 0.25, 0.8, 4, 4.7 μm, and (0.5, 6.5, 0.5, 45) μm, respectively. The SEM image of the waveguide facet is shown. The simulated transverse-electric optical mode profile at 3 μm is plotted.

Here, we present a monolithic optoelectronic platform in the MIR based on a suspended TFLN platform, capable of both high-speed amplitude and phase modulation (Fig. 1a). The air suspended TFLN is used to overcome the limitation of the absorption loss in the MIR from the underlying silicon dioxide layer as well as to support a tight optical confinement in the waveguide and reduce the MW propagation loss, both of which are critical to achieve a low switching voltage^10^. Co-designed with the optical waveguide, the segmented coplanar MW electrodes are deployed to further reduce the ohmic loss and achieve velocity matching between the MW and optical signals, supporting high-speed EO response. The demonstrated traveling-wave-based Mach-Zehnder amplitude modulator (AM) and double-pass phase modulator (PM) are based on (non-resonant) waveguide structures, which can operate in a broad MIR wavelength range and be compatible with integrated MIR light sources. Unlike electro-absorption or carrier-based modulators^32,33^, our modulator platform based on the Pockel’s effect not only allows for high-fidelity data links for free space optical communication via independent amplitude and phase modulation, but also can be used to generate broadband frequency-agile optical frequency comb^35^ for spectroscopy and sensing applications (Fig. 1b, c)^15^. Until now, none of the existing platforms has achieved broadband tunable MIR EO frequency comb generation^11,36,37^.

## Results and discussion

Figure 1d presents an optical microscope image of the integrated MIR modulator chip with a footprint of 25 × 7 mm^2^. The chip is fabricated on an 800-nm X-cut LN on 4.7 μm oxide on a Si substrate wafer. The waveguide has a top width of 4 μm and an etch depth of 500 nm, patterned via electron-beam lithography and ion milling (see “Methods”). Air holes are then defined and etched on the remaining 300-nm LN slab via a second step of lithography and etching, followed by the metal-electrode patterning, deposition and lift-off process. The periodic array of air holes, used to suspend the waveguide through wet undercut etching, has a pitch of 13.5 μm, dimensions of 3.5 × 1 μm^2^, and a center-to-waveguide distance of 5.75 μm. The air holes are placed in between the metal segments and used to selectively release the bottom oxide only around the photonic waveguide via a wet etching process, providing both good mechanical and thermal support for the electrodes. The scanning electron microscopy (SEM) image in Fig. 1e shows the structures of the air holes, suspended optical waveguide, and the segmented microwave electrodes. We optimize the operation for a fundamental transverse electric (TE) mode. Here, the smallest electrode gap of 6.5 μm along with the waveguide width of 4 μm is chosen to maximize the EO response as well as to minimize the metal-induced optical loss (see Supplementary Methods, Section S1.3). The cross-section of the EO waveguide and microwave transmission line is illustrated in Fig. 1f along with the optimized device geometry parameters after co-design and the simulated optical mode profile at 3 μm. Both the SEM image and the simulated optical mode profile shown in Fig. 1f correspond to the waveguide at the cleaved facet region used for edge coupling, where the waveguide width is approximately 2 μm. After input coupling, the waveguide width is gradually tapered to the designed width of 4 μm used in the active modulation section. The chip is cleaved for edge coupling at both input and output facets.

**Fig. 2:**
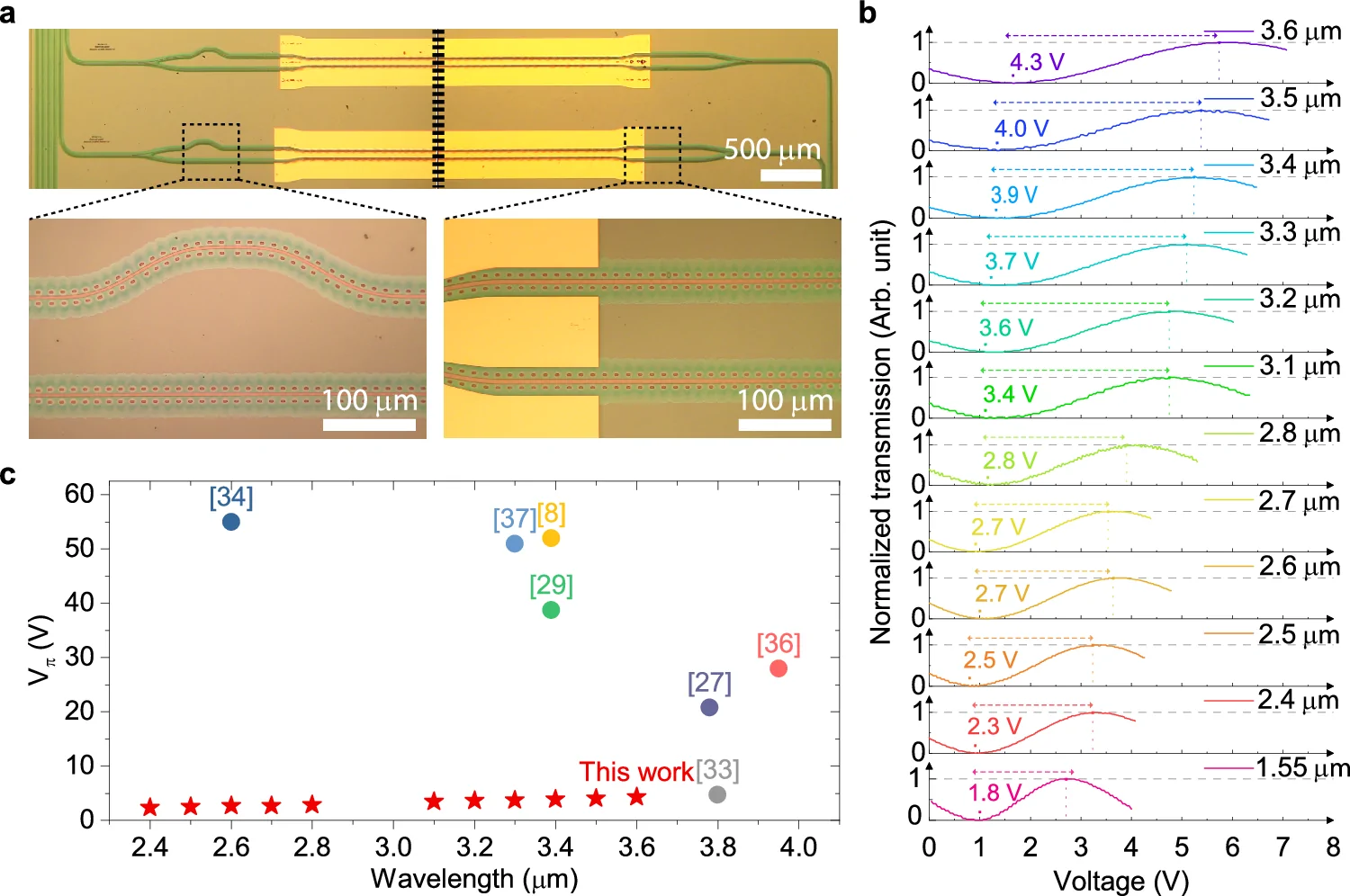
Characterization of the near-DC half-wave voltage *V*_*π*_ of a 2-cm-long air-suspended amplitude modulator. **a** Optical microscope images. The two optical paths in the amplitude modulator have a length difference of 21 μm, enabling bias-point tuning by varying the optical wavelength. **b** Normalized optical transmission as a function of applied voltage at different MIR wavelengths and at 1.55 μm in the NIR. The measured half-wave voltage at 100 kHz ranges from 2.3–4.26 V over the wavelength range from 2.4 to 3.6 μm, showing an approximately linear dependence on the operating wavelength. The suspended amplitude modulator also operates over more than one octave of optical bandwidth, with a half-wave voltage of 1.8 V at 1.55 μm, corresponding to *V*_*π*_*L* = 3.6 V·cm. **c** Comparison of *V*_*π*_ with those of other MIR electro-optic platforms. The LN amplitude modulator exhibits the lowest *V*_*π*_, with *V*_*π*_*L* values of 4.6 V·cm at 2.4 μm and 8.6 V·cm at 3.6 μm.

We first characterize the near-DC half-wave voltage, namely *V*_*π*_, of our AM device at a low modulation frequency of 100 kHz. The suspended AM shown in Fig. 2a includes two Y-shape splitters, two asymmetric arms of a 21 μm length difference and an active modulation length of 2 cm in a push-pull configuration. The continuous-wave tunable optical parametric oscillator (Toptica, TOPO) is used to inject the device via a free space objective lens at a MIR wavelength range from 2.4–3.6 μm. We record the modulated optical output using an AC-coupled mercury cadmium telluride (MCT) detector while applying a saw-tooth voltage waveform to the electrodes at 100 kHz. Figure 2b plots the optical transmission as a function of applied voltage at various MIR wavelengths, featuring the *V*_*π*_ of 2.3 V at 2.4 μm to 4.3 V at 3.6 μm. The corresponding half-wave voltage length product (*V*_*π*_*L*) is 4.6 V⋅cm at 2.4 μm and 8.6 V⋅cm at 3.6 μm wavelength, respectively. The *V*_*π*_ scales near linearly with the optical wavelength *λ*, considering that the accumulated phase shift *Δϕ* ∝ *Γ*/*λ* where *Γ* is the mode overlap (see Supplementary Methods, Section S1.1). As the suspended optical waveguide supports guided mode over an ultrabroad bandwidth, we test the *V*_*π*_*L* at the NIR wavelength of 1.55 μm to be 3.6 V·cm, demonstrating our integrated EO modulator operation across a 1.2-octave span. Here, the reported *V*_*π*_*L* values are based on a Mach-Zehnder-interferometer AM operated in a push-pull configuration. The slightly higher *V*_*π*_*L* observed at longer wavelengths is consistent with the expected wavelength scaling of electro-optic phase accumulation, where reduced optical wave vector and increased optical mode size lead to lower modulation efficiency. The extinction ratio is measured to be 7 dB in the MIR using a lock-in amplifier and can be improved by deploying a single-mode waveguide at the Y splitter to suppress the scattering into undesired optical modes (see Supplementary Methods, Section S1.2). We compare the near-DC *V*_*π*_ with the state-of-the-art reported in other MIR modulators at a similar wavelength range in Fig. 2c. This work presents the lowest *V*_*π*_ ever reported in the MIR, reaching CMOS-compatible voltages, and the broadest MIR optical bandwidth, both of which are critical for in-parallel power-efficient modulation.

**Fig. 3:**
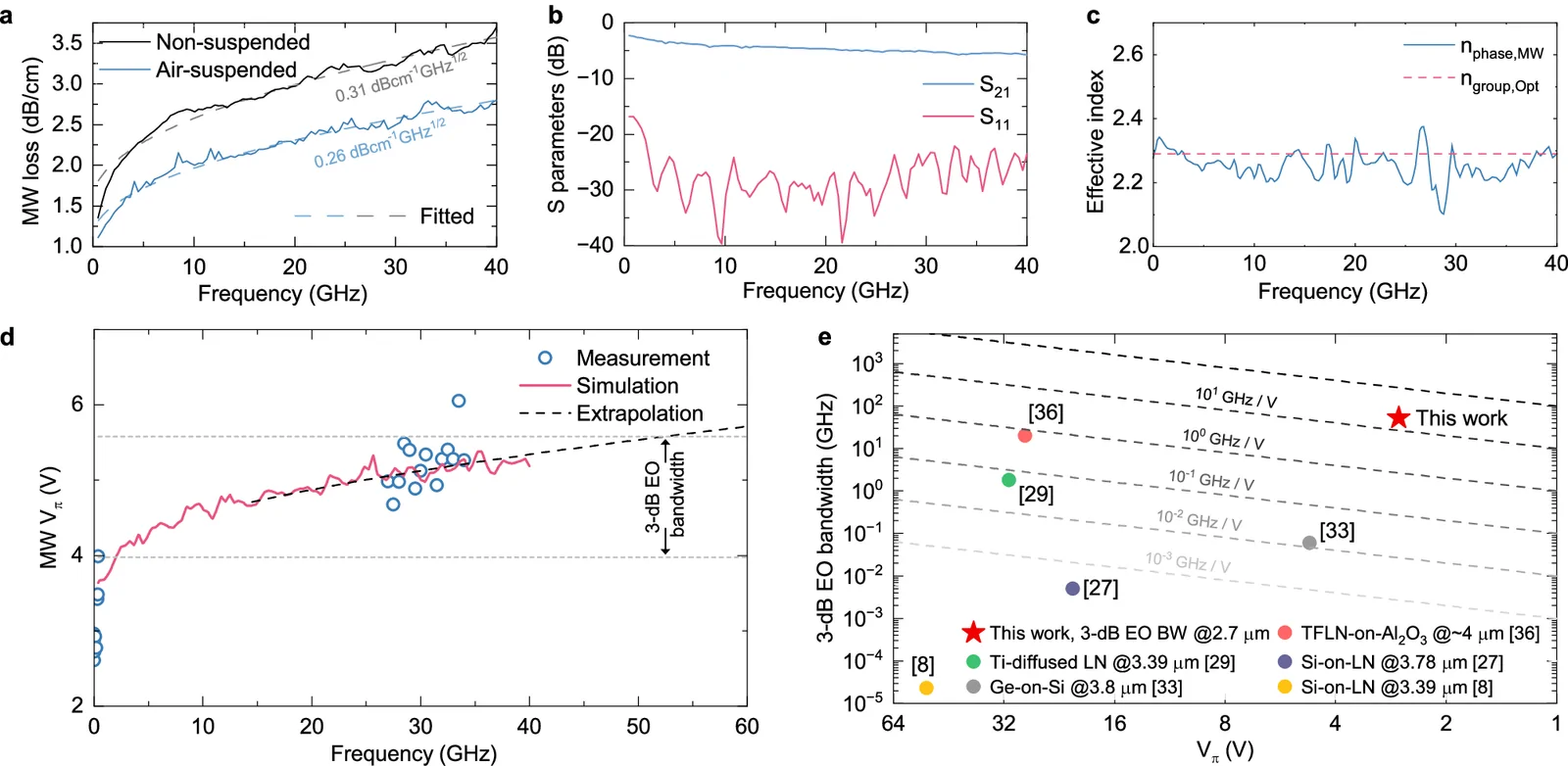
Characterization of the MIR modulator up to 40-GHz microwave (MW) frequencies. **a** Measured microwave loss on the segmented co-planar electrodes before and after air suspension. Segmented travel-wave electrodes combined with air suspension lead to a reduced MW propagation loss of 2.75 dB/cm at 40 GHz and an ohmic-loss-limited slope of 0.26 dB/cm/GHz^0.5^, which is comparable to the loss slope of the best NIR LN modulators reported^43^. **b** Measured the electrical transmission *S*_21_ and reflection *S*_11_ spectrum. The measured reflection is below −20 dB from 2–40 GHz range, indicating a well-matched impedance to 50 *Ω*. **c** Measured MW phase index, which matches well with the optical group index based on the air-suspended TFLN waveguide (dashed line). **d** MW *V*_*π*_ measured from 100 kHz to 34 GHz. MW *V*_*π*_ values are measured to be 5 V at 27 GHz and 5.26 V at 34 GHz for a 2-cm-long EO AM at the optical wavelength of 2.7 μm. The MW *V*_*π*_ is extracted via the optical spectrum from the AM under MW driving. The measurement results match the simulation based on the measured S parameters in (**b**). A rapid low-frequency roll-off is observed due to impedance mismatch. Therefore, the EO response is referenced to the measured response at 0.4 GHz, yielding an extracted 3-dB EO bandwidth of 52 GHz (dashed line). The electro-optic response scales inversely with ${V}_{\pi }^{2}$ and decreases by only 2.3 dB at 40 GHz, the frequency limit of our signal generator. **e** Comparison of the modulator figure of merit, defined as the ratio between the 3-dB EO bandwidth (BW) and the near-DC *V*_*π*_. The AM in our work demonstrates significantly higher BW/*V*_*π*_ values of 19.3 GHz/V, as compared to the literature values (the dashed lines indicate constant BW/*V*_*π*_ values).

Next, we demonstrate the high performance of the suspended MIR modulator at microwave frequencies, including the EO response and the MW *V*_*π*_*L*. We first measure the microwave attenuation loss, reflection loss and phase index of the segmented electrodes via a vector network analyzer in a frequency span from 10 MHz to 40 GHz, shown in Fig. 3a–c. The MW loss is extracted from *S*_21_ and measured to be 2 dB/cm at 10 GHz and 2.75 dB/cm at 40 GHz, respectively. The fitted loss slope coefficient is 0.26 dB/cm/GHz^0.5^ after air suspension (Fig. 3a). We discover a higher MW loss slope of 0.31 dB/cm/GHz^0.5^ and an increased loss of 3.75 dB/cm at 40 GHz before air suspension of the waveguide (Fig. 3a), which suggests a potential loss channel induced by the bottom oxide layer. In conclusion, the record MW loss is a result of a thick Au metal thickness of 800 nm, optimization of the slow-wave T-shaped segment design, and removal of the oxide layer. Secondly, Fig. 3b also plots the reflection *S*_11_ below -20 dB across the 2–40 GHz range, indicating that the characteristic impedance of the transmission line is well matched to 50 Ω as designed. At last, Fig. 3c shows that the MW phase index, obtained by *n*_*phase*,*MW*_ = (*c*_0_ × group delay)/ (electrode length), matches well with the optical group index (*n*_*grp*,*optical*_) of 2.29. The achieved high-performance electrodes contribute to a lower MW *V*_*π*_ as the electrode length increases. The MW *V*_*π*_ is extracted from the optical spectrum of the driven modulator output measured by a Fourier-transform infrared spectrometer. High-speed photodetectors operating beyond 3 μm with bandwidths exceeding tens of gigahertz are currently limited in availability, and therefore, the electro-optic response is characterized using optical modulation sidebands measured in the spectral domain^38,39^. Figure 3d plots the measured MW *V*_*π*_ of our 2-cm-long AM to be 4.5–6.5 V at 27–34 GHz frequency, which agrees well with our simulation based on the device parameters. The *V*_*π*_ from 100 kHz to 0.4 GHz is measured by the MIR photodetector and also plotted. The direct *V*_*π*_ measurement between 0.4 GHz and 27 GHz is limited by the available RF amplifier frequency bands and the 7.5-GHz spectral resolution of the Fourier-transform optical spectrum analyzer (Thorlabs OSA205C), since the generated optical sidebands at these frequencies become insufficiently separated to be reliably resolved by the spectrometer. The EO response can be simulated from the measured MW properties and scales inversely with the square of the microwave *V*_*π*_, which matches well with the experimental data (Fig. 3d). It is referenced to a measured non-zero frequency of 0.4 GHz, corresponding to the highest frequency that can be reliably measured with our mid-infrared photodetector before the onset of the impedance-mismatch-induced low-frequency roll-off^40–42^, while capturing the intrinsic traveling-wave microwave response^42–44^. The EO response decreases by 2.3 dB at a MW frequency of 40 GHz, which is the frequency limit of our signal generator. By fitting the measured response and referencing the low-frequency (0.4 GHz) response level, the extracted 3-dB EO bandwidth is estimated to be 52 GHz(dashed line). To the best of our knowledge, this is the first time that the *V*_*π*_ has ever been measured at tens of GHz frequency range and presents the broadest EO bandwidth demonstrated so far in any MIR modulators. To note, another commonly used figure-of-merit (FOM) is the ratio between the 3-dB electro-optic bandwidth and the near-DC *V*_*π*_ (BW/*V*_*π*_), which provides a practical performance metric for traveling-wave modulators operating near microwave-optical velocity matching, where the response is primarily limited by microwave attenuation^38^. This metric is used here as an empirical benchmark for comparison with prior works rather than as a universal physical scaling law. In our work, this FOM is achieved at 19.3 GHz/V, orders of magnitude larger than the reported value (Fig. 3e).

**Fig. 4:**
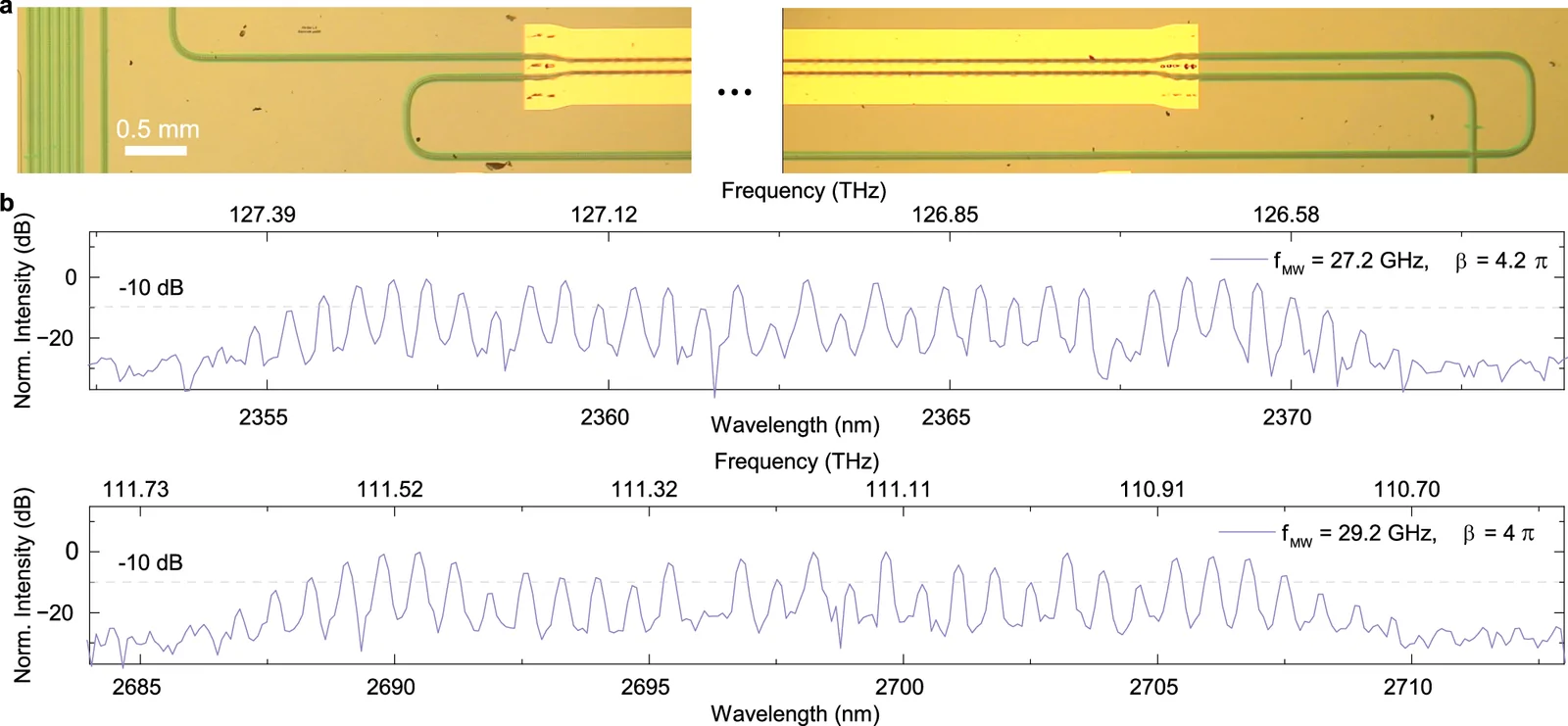
Frequency-agile MIR electro-optic frequency combs based on an integrated recycled phase modulator. **a** A microscopic image of the suspended double-passing phase modulator with the total modulation length of 4 cm. **b** Broadband EO frequency comb generation using the same PM device at two different MIR wavelengths of 2.36 μm and 2.7 μm. Total modulation indexes (*β*) of 4.2 *π* and 4 *π* at MW driving frequencies (*f*_MW_) of 27.2 and 29.2 GHz were measured at 2.36 μm and 2.7 μm pump wavelength, respectively. This corresponds to a 10-dB optical bandwidth of 0.8 THz.

In addition to the amplitude modulation, we demonstrate a suspended 2-cm-long TFLN PM and generate frequency agile EO frequency combs in the MIR. Figure 4a shows the optical image of the fabricated PM, where we adopt a double-pass photonic structure where the waveguide is rerouted into the same transmission line after a waveguide crossing. Therefore, the total active modulation length is achieved at 4 cm. Further leveraging the low MW *V*_*π*_ and large EO bandwidth of our platform, we generate an integrated MIR EO frequency comb with a 10-dB optical bandwidth of 0.8 THz (20 nm) and a total comb line number of 33 at a center wavelength of 2.7 μm by driving the PM with a single-frequency MW signal at 29.2 GHz (Fig. 4b). In addition, by driving the same device, we achieve an EO comb spectrum at 2.36 μm with a 27.2-GHz line spacing and a similar 10-dB span of 0.8 THz (15 nm), which shows the frequency agility of the non-resonant waveguide-based MIR EO comb generator in both center wavelengths and comb line spacings. The total modulation index (*β*) we achieve is 4.2 *π* and 4 *π* at 27.2 GHz and 29.2 GHz, respectively. The current comb spectrum is generated using a phase-modulation-only configuration, and the spectral envelope is therefore not actively flattened. Improved spectral flatness can be achieved by incorporating amplitude modulation or time-lens techniques prior to phase modulation, as demonstrated in prior work and currently under investigation^35,45–48^. This is the first demonstration of integrated EO comb generation in the MIR with tens of GHz repetition rates, without any need for parametric conversion. Complementary to the semiconductor-based combs^1,49,50^ and Kerr microcombs in the MIR^51,52^, the EO comb sources offer unique advantages, including tunable spectral resolution, wavelength multiplexing and robust mode-locking operation for sensing and communication applications^9^.

**Fig. 5:**
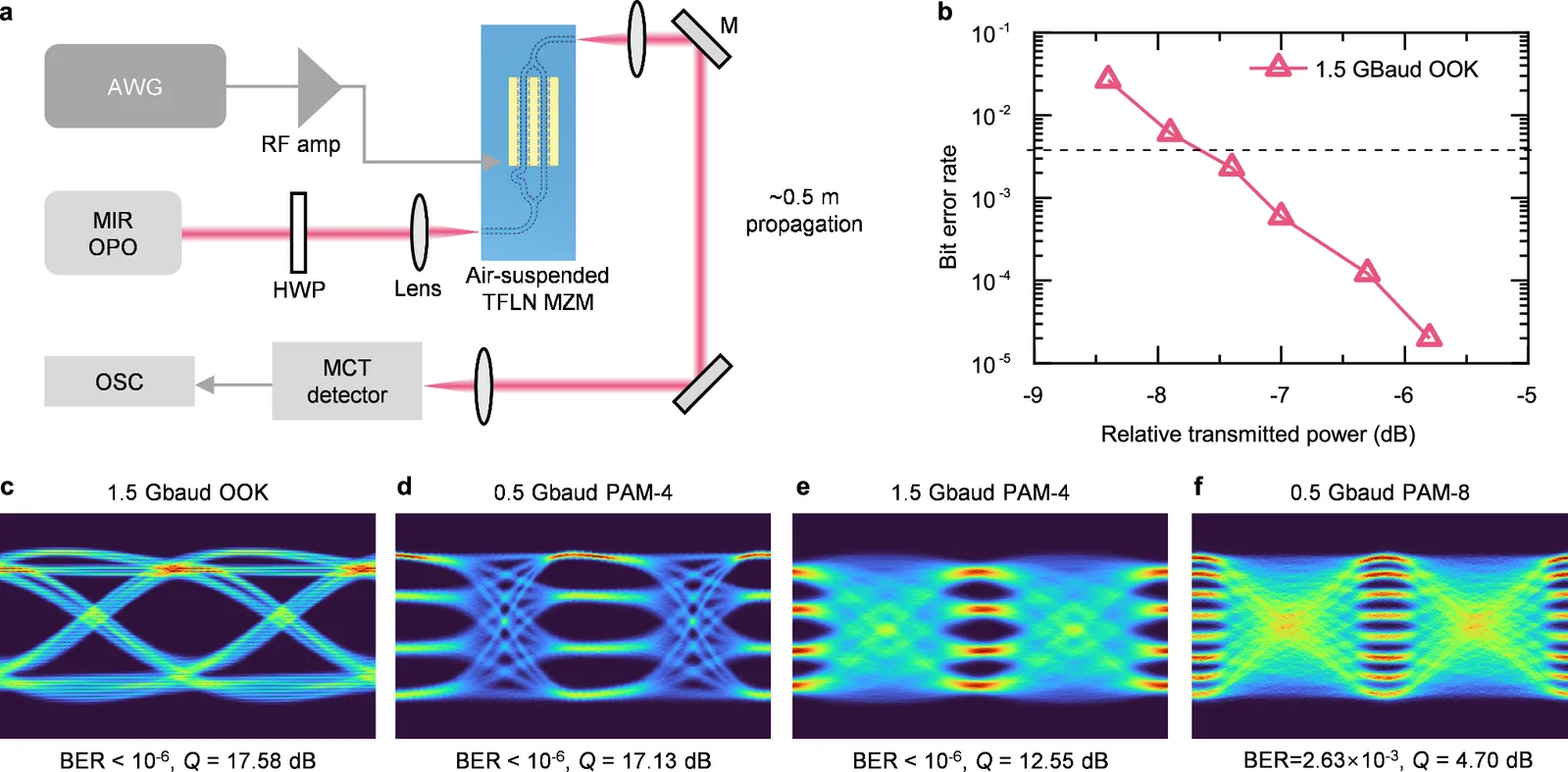
Experimental MIR communication link using an air-suspended TFLN intensity modulator. **a** Experimental setup for measuring the communication link at 2.7 μm wavelength. OPO optical parametric oscillator, HWP half-wave plate, AWG arbitrary waveform generator, MCT detector mercury cadmium telluride detector, DSO digital storage oscilloscope. **b** Measured BER curve versus transmitted optical power for 1.5-Gbaud on-off keying (OOK) signal. **c**–**f** Experimental results of eye diagram, BER values, and Q-factors of 1.5-Gbaud OOK, 0.5-Gbaud pulse amplitude modulation (PAM)-4, 1.5-Gbaud PAM-4, and 0.5-Gbaud PAM-8. The achievable data rate is limited by the 3-dB bandwidth of our MIR detector of 1 GHz.

Finally, we evaluate a free-space communication link based on the suspended TFLN AM as a proof-of-principle demonstration. We experimentally demonstrate an intensity modulation/direct detection (IM/DD) MIR link at 2.7 μm. Figure 5a illustrates the experimental setup (see “Methods”). The modulated MIR output propagates 0.5 m in the free space and is detected by the MCT detector with a 3-dB bandwidth of >1 GHz. We first measure the bit error rate (BER) performance of OOK signals at various input power levels to the AM with a baud rate of 1.5 Gbaud, as shown in Fig. 5b. The reference case (0 dB) corresponds to the maximum transmitted power. In this setup, the system performance is primarily limited by the detector bandwidth and total loss, resulting in a BER decrease from 10^−2^ to 10^−5^ within a 2.5-dB power range. Additionally, we demonstrate higher-order intensity modulation formats at a maximum transmitted power, including 4-level and 8-level pulse amplitude modulation (PAM-4 and PAM-8). Eye diagrams, BER values, and Q-factors for different modulation formats and baud rates are presented in Fig. 5c–f for comparison. All configurations achieved BER values below the 7% hard-decision forward error correction (HD-FEC) threshold. The phase modulator demonstrated on the same platform further highlights its capability for phase-modulated formats and more complex data modulation.

In conclusion, the suspended TFLN EO devices demonstrated here are prototypical building blocks for a new generation of MIR integrated photonic architectures, expanding functionality beyond amplitude modulation to include phase modulation and frequency-comb generation. We achieved record-low *V*_*π*_ at both near-DC and previously unexplored tens-of-GHz microwave frequencies range, a broad EO bandwidth exceeding 40 GHz, and an ultra-wide optical window covering 1.55 μm and 2.4–3.6 μm in the MIR. This work marks the first time that an MIR EO modulator exceeds the centimeter-scale interaction length while achieving a modulation index exceeding 4*π* and a FOM of 19.3 GHz/V for the EO bandwidth per drive voltage, more than an order of magnitude improvement over other MIR platforms (Table 1). Leveraging these unique characteristics, we demonstrated the first, to our knowledge, direct EO frequency comb generation in the MIR with a 0.8-THz span and over 33 comb lines. These results establish TFLN as a compelling monolithic platform for scalable MIR EO systems. The suspended platform could be extended to operate beyond 3.6 μm to explore the full LN transparency window (up to 5 μm) by increasing the electrode gap and applying the same co-design principle. The optical loss can be improved via further optimizing the facet design and fabrication process with thermal and chemical treatment^53–55^. In addition, recent studies suggest that ultrathin plasma-enhanced atomic layer deposited passivation coatings^56^ could potentially improve the environmental robustness and long-term stability of the mid-IR electro-optic modulators. A straightforward yet crucial next step will be to integrate in-phase/quadrature (IQ) modulator configurations for coherent MIR communication and vector signal processing. We highlight the versatility of the TFLN platform, where periodically poled TFLN waveguides would further be integrated to down-convert the high-speed MIR signal to the NIR as the receiver of an FSOC link. Further integration of the AM and PM on the same chip will also enable MIR pulse generation and on-chip waveform synthesis^35^. The demonstrated comb bandwidth of 0.8 THz would lead to a 1.7-ps pulse train at 2.7 μm at a 30-GHz repetition rate via EO mode-locking. On-chip EO modulation offers a path to stabilized MIR comb sources and synchronized pulse trains^35,57^, bridging the gap between semiconductor lasers and electro-optic frequency synthesis. With continued advancement of QCLs and chip-based parametric oscillators^11,58^, we envision a hybrid passive and active nonlinear integrated system where frequency conversion, EO modulation and comb generation could coexist. Such systems would not only enable spectrally tailored dual-comb spectroscopy for molecular sensing and metrology^59^ but also provide multi-wavelength coherent sources for free-space optical communication^3,23,60^ and astronomical spectrograph calibration^61,62^. The ability to combine efficient electro-optic modulation, broadband MIR transparency, and scalable chip-level integration marks an important step toward fully reconfigurable, multifunctional MIR photonic systems.

**Table 1:** Comparison of mid-infrared electro-optic modulators

Wavelength (μm)	Platform design	Scheme	Function	L (cm)	*V*_*π*,*DC*_(V)	*V*_*π*,*MW*_(V)	Optical loss (dB/cm)	MW loss (dB/cm)	EO BW	EO BW per *V*_*π*,*DC*_ (GHz/V)	Application	Ref.
2.4–3.6	Air-suspended TFLN, MZI	Pockels	AM, PM, combs	2	2.3–4.2	4.5–6.5^*b*^	2.2–2.8	2.75 @40 GHz	40 GHz^*c*^, 52 GHz^*d*^	19.3	EO comb 0.8-THz span, Mod index of 4*π,* OOK@1.5 Gbaud, PAM8@ 0.5 Gbaud	This work
2.5	TiO_2_-on-LN, WG	Pockels	AM	NA	NA	NA	NA	NA	NA	NA	NA	^28^
2.6	BTO-on-oxide, WG	Pockels	AM	0.2	55	NA	2.3	NA	NA	NA	NA	^34^
3.39	Si-on-LN, MZI	Pockels	AM	0.5	52	NA	2.5	NA	>23 kHz^*a*^	4.4 × 10^−7^	NA	^8^
3.39	Ti-diffused LN, MZI	Pockels	AM	0.8	31	NA	NA	NA	1.8 GHz	0.058	NA	^29^
3.78	Si-on-LN, MZI	Pockels	AM	0.6	20.8	NA	4.5	NA	>5 MHz^*a*^	0.00024	NA	^27^
3.8	Si-on-SiO_2_, MZI	TO	AM	NA	NA	NA	3.5	NA	23.8 kHz	NA	NA	^26^
3.85–4.1	BP-Si-on-SiO_2_, WG	EA	AM	NA	NA	NA	NA	NA	>60 kHz	NA	NA	^30^
3.8	Ge-on-Si, MZI/EAM	Cl/CA	AM	0.1	4.7	NA	NA	NA	>60 MHz	0.013	OOK@60 MHz	^33^
3.72–3.8	Si-on-oxide, MZI/EAM	Cl/CA	AM	NA	NA	NA	11.5	NA	NA	NA	OOK@125 Mbit/s	^31^
3.95–4.3	TFLN-on-Al_2_O_3_, MZI	Pockels	AM	0.8	28	NA	2.2	NA	20 GHz	0.71	OOK@10 Gbit/s	^36^^*e*^
3.3–3.8	Air-clad TFLN, cavity	Pockels	AM	0.15	51	NA	4	NA	NA	NA	NA	^37^

*ER* extinction ratio; *EO* electro-optic; *TO* thermo-optic; *EA* electro-absorption; *BP* black phosphorus; *EAM* electro-absorption modulator; *CI* free-carrier injection; *CA* free-carrier absorption; *Mod* modulation.

^a^ The measured bandwidths are limited by the photodetector speed.

^b^*V*_*π*_ was measured at 2.7 μm and microwave frequencies from 25 to 35 GHz.

^c^ 2.3 dB EO bandwidth, limited by the signal generator.

^d^ Extrapolated value.

^e^ During the preparation of this manuscript, related work was posted online; its reported performance is included in this table.

## Methods

### Device fabrication

To support guided modes in the MIR spectrum, we patterned waveguides using electron beam (e-beam) lithography with a thick hydrogen silsesquioxane (HSQ) resist and dry etched 500 nm of lithium niobate (LN) using reactive ion etching (RIE). Air holes were patterned near the LN waveguides using direct laser writing and etched with RIE through a photoresist mask. Subsequently, 800 nm-thick electrodes were patterned with e-beam lithography, followed by metal deposition using a thermal evaporator and a lift-off process. The underlying SiO_2_ layer was then etched using a buffered oxide etch (BOE) solution.

### Communication setup

A TOPTICA continuous-wave (CW) optical parametric oscillator (OPO) is utilized in our system as a mid-infrared (MIR) source. Using a 1064 nm CW pump laser, this OPO can generate tunable ~1W MIR power within the range of 2.5–4 μm with a laser linewidth of approximately 500 kHz. The free-space output of the OPO is coupled into the integrated Mach-Zehnder amplitude modulator (AM) using a MIR collimating lens.

An arbitrary waveform generator (AWG) with a sampling rate of 20 GSa/s, followed by an electrical amplifier with 26 dB gain, is used to apply various intensity modulation signals to the AM. The electrical signal is delivered to the device electrode through a 40 GHz-bandwidth RF probe. After free-space propagation of approximately 0.5 m, the output of the modulator is focused onto a MIR mercury cadmium telluride (MCT) detector for detection. This MCT detector has a bandwidth of about 1 GHz.

The detector output is recorded by a digital storage oscilloscope (DSO) for data recovery. Specifically, offline digital signal processing (DSP) is applied to compensate for system degradation in multi-level pulse amplitude modulation (PAM) signals. A 20-tap adaptive filter based on recursive least-squares (RLS) equalization with a training sequence is used to improve signal quality. No additional DSP equalization is applied to the on-off keying (OOK) signal in this measurement.

## Supplementary information

Supplementary Information

Peer Review file

## Data Availability

The data supporting the findings of this study are available from the corresponding author upon request. Requests will be considered subject to applicable institutional and confidentiality requirements.
