# Supplementary material for: Suspended thin-film lithium niobate modulator for broadband mid-infrared light modulation and frequency comb generation
Source: Nat Commun. 2026 Sep 17;17:10936. doi: 10.1038/s41467-026-77674-y (PMC13680620; doi:10.1038/s41467-026-77674-y)
Supplement: Supplementary file 1 [file 41467_2026_77674_MOESM1_ESM.pdf]

Supplementary Information

Suspended thin-film lithium niobate modulator for broadband mid-infrared light modulation and frequency comb generation

Chun-Ho Lee*[1,2], Xinyi Ren*[1,2], Xinzhou Su*[2], Wonho Lee[3], Zile Jiang[2], Yue Yu[1,2,7], Huibin Zhou[2], Yue Zuo[2], Shaoyuan Ou[2], Reshma Kopparapu[1,2], Adam T. Heiniger[4], Moshe Tur[5,6], Alan E. Willner[2], Zaijun Chen[1,2], and Mengjie Yu†[1,2,7]

[1]Department of Electrical Engineering and Computer Sciences, University of California, Berkeley, CA, USA
[2]Ming Hsieh Department of Electrical and Computer Engineering, University of Southern California, Los Angeles, CA, USA
[3]PHY Research Lab, Intel Labs, Hillsboro, OR, USA
[4]TOPTICA Photonics Inc., Pittsford, NY 14534, USA
[5]School of Electrical Engineering, Tel Aviv University, Ramat Aviv 69978, Israel
[6]Faculty of Electrical Engineering, Holon Institute of Technology, Holon 58102, Israel
[7]Materials Sciences Division, Lawrence Berkeley National Laboratory, Berkeley, California 94720, USA

Contents

S1 Supplementary Methods **2**
S1.1 Wavelength dependent near-DC V_π 2
S1.2 Extinction ratio analysis 3
S1.3 Insertion Loss Analysis 4

*These authors contributed equally.
†Corresponding author: mengjie.yu@berkeley.edu

S1 Supplementary Methods

S1.1 Wavelength dependent near-DC V_π

$$\Delta\beta_{\mathrm{TE}} = \frac{2n_e\,\Delta n_e}{2\beta_{\mathrm{TE}}}k_0^2 = -\frac{n_e^4 r_{33}\Gamma_{\mathrm{mo}}}{2\beta_{\mathrm{TE}}}k_0^2 \tag{S1}$$

$$V_\pi \cdot L = \frac{\pi}{\Delta\beta_{\mathrm{TE}}} \times 100\ (\mathrm{V\cdot cm}) \tag{S2}$$

Definitions:

- $\Delta\beta_{\mathrm{TE}}$: total phase change per meter per volt for TE guided mode.
- k_0: wavevector in vacuum.
- β_{TE}: propagation constant of the guided mode.
- n_e: extraordinary refractive index of $LiNbO_3$ (LN).
- r_{33}: electro-optic (EO) coefficient.
- Γ_{mo}: mode overlap factor.

Dispersive DC V_π is studied using COMSOL finite element method numerical simulation using equation (1) and (2). Supplementary Figure 1a shows comparison between measurement and simulation results. Trenching effect at the bottom of waveguide sidewall is included in the simulation. Measured DC V_π values matches well to the simulation in all wavelength ranges.

We note that a higher V_π is expected at longer optical wavelengths due to both the reduced optical wave vector ($k = 2\pi/\lambda$), which slows phase accumulation per unit length, and the increased optical mode size, which decreases the microwave–optical mode overlap (Supplementary Fig. 1b).

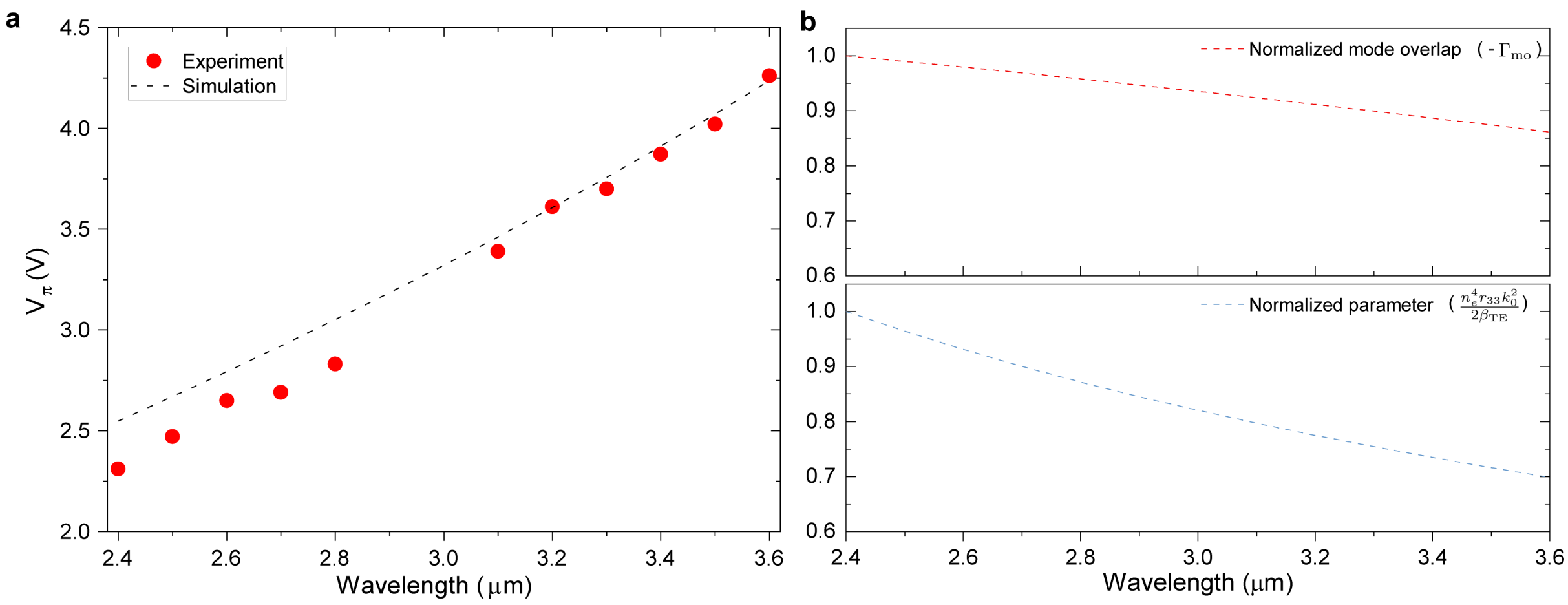

Supplementary Figure 1: Analysis for wavelength-dependent DC V_π.
a, DC V_π measurement and simulation for the wavelength range of 2.4–3.6 μm. **b**, Simulated mode overlap factor and other parameters used for $\Delta\beta_{\mathrm{TE}}$ calculation.

S1.2 Extinction ratio analysis

The extinction ratio is measured using a chopper, a MIR photodetector (PVI-4TE-8), and a lock-in amplifier as the AWG sweeps the applied voltage on the AM. After the sinusoidal fitting, the extinction ratio is 7.1 dB based on the maximum and minimum transmission values (Supplementary Fig. 2). The extinction ratio is primarily limited by the higher-order mode coupling to the slab at the second Y-splitter/merger, which can be improved by using a single-mode waveguide width of 0.9 µm instead of 2 µm in our case.

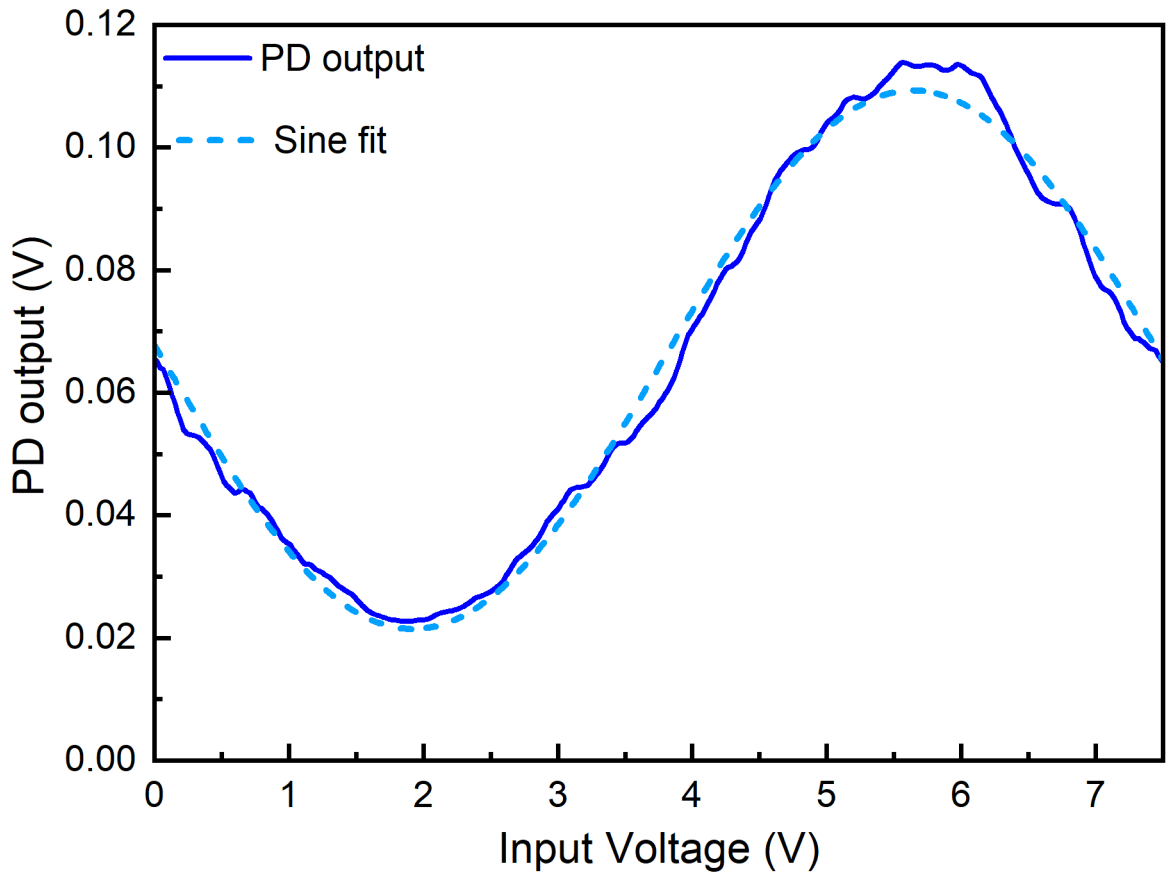

Supplementary Figure 2: Extinction measurement of the air-suspended amplitude modulator at 2.7 µm wavelength.

S1.3 Insertion Loss Analysis

We use two free-space objective lenses (Thorlabs, C036TME-E) to couple the MIR light into and out of the devices over a wavelength range of 2.4–3.6 µm, with an off-chip optical power ranging from 60 to 700 mW at the chip facet. Supplementary Table 1 summarizes the optical losses measured from both the suspended device used in the main manuscript and non-suspended reference devices (air clad) of identical dimensions. The passive and active propagation losses were extracted from waveguides, AMs and recycled PMs with different waveguide lengths. As shown in the supplementary table 1, the optical loss at 2.4 µm remains comparable before and after suspension, while the suspended devices exhibit noticeably lower loss at 2.7, 2.8 and 3.6 µm compared to the non-suspended counterpart. This result agrees well with the spectral absorption feature of SiO_2 in the mid-infrared where the absorption has a peak around 2.7-2.8 µm and increases starting at 3.1 µm. Even though our device is designed and measured up to 3.6 µm, the suspended TFLN approach is expected to continuously reduce the propagation loss beyond 3.6 µm up to the end of the transmission window of LN at 4.5-5 µm.

However, the active waveguides, which include nearby metal electrodes for electro-optic modulation, show higher loss than the passive sections. The measured active propagation losses at 2.4 µm and 3.6 µm are 6.4 dB/cm and 10.5 dB/cm, respectively. The exact cause of this additional attenuation is still under investigation as the simulated metal induced loss is 0.2 and 4.8 dB/cm respectively. One of the possible reason is the high energy electron injection during the ebeam lithography of the metal electrodes nearby the waveguide which induces higher free carriers loss at higher optical wavelength, which could be improved in the future via controlled annealing condition and photolithography.

Supplementary Table 1. Measured propagation loss of the fabricated devices.

	**Wavelength** (µm)	**Propagation loss passive (dB/cm)**
**Suspended**		
	2.4	2.2
	2.7	1.2
	2.8	4.6
	3.6	2.8
**Non-suspended**		
	2.4	2.4
	2.7	7.2
	2.8	11.4
	3.6	4.3
