# Supplementary material for: Suspended thin-film lithium niobate modulator for broadband mid-infrared light modulation and frequency comb generation
Source: Nat Commun. 2026 Sep 17;17:10936. doi: 10.1038/s41467-026-77674-y (PMC13680620; doi:10.1038/s41467-026-77674-y)
Supplement: Supplementary file 2 [file 41467_2026_77674_MOESM2_ESM.pdf]

nature portfolio

Peer Review File

Suspended thin-film lithium niobate modulator for broadband mid-infrared light modulation and frequency comb generation

Corresponding Author: Professor Mengjie Yu

This file contains all reviewer reports in order by version, followed by all author rebuttals in order by version.

Attachments originally included by the reviewers as part of their assessment can be found at the end of this file.

Version 0:

Reviewer comments:

Reviewer #1

(Remarks to the Author)

(Remarks on code availability)
Please, see the attached document

Reviewer #2

(Remarks to the Author)
I co-reviewed this manuscript with one of the reviewers who provided the listed reports. This is part of the Nature Communications initiative to facilitate training in peer review and to provide appropriate recognition for Early Career Researchers who co-review manuscripts.

(Remarks on code availability)

Reviewer #3

(Remarks to the Author)
The work represents one of the first long wavelength electro optic modulators - if not the first - and as such has a respectable speed and voltage requirement when compared to alternative approaches at this wavelength. A comprehensive set of experiments give clarity on the new opportunities that may be foreseen.
The absense of good modulators in the mid infrared limits a wide range of use cases for integrated photonics, so this will be important for sensing and free space communications research.
The work is carefully specified for mid infrared where there is limited prior art. Therefore the work is self consistent. The methods are sound. The only unaddressed question would be why the performance in mid infra red is not as good as near infra red in terms of bandwidth and efficiency. It would be of interest to know whether the authors has insight on this.

(Remarks on code availability)
no comment

Reviewer #4

(Remarks to the Author)
This manuscript reports a suspended thin-film lithium niobate (TFLN) electro-optic platform for mid-infrared (MIR) applications, demonstrating low half-wave voltage, broadband operation, and high electro-optic (EO) bandwidth. The authors further present an MIR EO frequency comb and a proof-of-concept free-space optical communication link. The work addresses an important challenge in MIR photonics and presents a promising device platform combining low drive voltage and high-speed operation. The manuscript is also well written. I recommend its publication after some revisions. My detailed

comments are listed below.
1. While the authors claim “low-loss EO modulation”, this is not sufficiently supported by the experimental results. For example, the measured active propagation loss at 3.6 μm is as high as 10.5 dB/cm. This result significantly weakens the claim of “low-loss EO modulation”.
2. Characterizing the bandwidth of a modulator typically requires a high-speed photodetector (PD). However, this approach is not adopted in this work. I guess the reason is the lack of commercially available high-speed PDs at these wavelengths. If so, the authors should clarify this point. On the other hand, commercial high-speed PDs are available around 2.6 μm. The authors may consider characterizing the modulator’s bandwidth at 2.6 μm using a commercial high-speed PD. In addition, the 3-dB bandwidth is referenced to the performance at 2 GHz, which is not commonly seen in the literature. It is recommended to define the bandwidth relative to the near-DC response (e.g., kHz or MHz).
3. The authors provided several design parameters, such as the metal gap and waveguide height. However, details regarding the air holes are missing. For example, the hole size, hole pitch, and distance from the waveguide core are not provided. These parameters should also be included.
4. Figure 4(b): While a 0.8-THz-span MIR EO comb with more than 30 lines is interesting, the results shown in Fig. 4(b) appear preliminary, particularly given the large power variation among the comb lines. It is not clear what practical applications can be enabled by this MIR EO comb.
5. Figure 1(f): The claimed waveguide width (w0) of 4 μm is not consistent with the two scale bars in this figure. The authors should double-check the scale bars in all figures.
6. Figure 5(a): Please check the use of “OSC” in the figure and “DSO” in the caption for consistency.
7. Table 1: Please check whether “PAM8@1.5 Gbaud” is correct.

(Remarks on code availability)

Reviewer #5

(Remarks to the Author)
I co-reviewed this manuscript with one of the reviewers who provided the listed reports. This is part of the Nature Communications initiative to facilitate training in peer review and to provide appropriate recognition for Early Career Researchers who co-review manuscripts.

(Remarks on code availability)

Version 1:

Reviewer comments:

Reviewer #1

(Remarks to the Author)
Both the main reviewer the co-reviewer reviewed the author's response and we think that our questions and concerns were properly addressed. We think that the paper is fit to be published.

(Remarks on code availability)

Reviewer #2

(Remarks to the Author)
I co-reviewed this manuscript with one of the reviewers who provided the listed reports. This is part of the Nature Communications initiative to facilitate training in peer review and to provide appropriate recognition for Early Career Researchers who co-review manuscripts.

(Remarks on code availability)

Reviewer #4

(Remarks to the Author)
The authors have addressed most of my concerns, but some new concerns have arisen after reading the revised manuscript and the rebuttal letter. Before publication, the authors should address the following concerns.

1. In the rebuttal letter, the authors claim that “the EO roll-off is often defined relative to a low but non-zero reference frequency, typically 1-2 GHz”, and refer to these references:
[1] P. Kharel et al., Breaking voltage–bandwidth limits in integrated lithium niobate modulators using micro-structured electrodes, Optica 8, 357-363 (2021). doi: 10.1364/OPTICA.416155
[2] M. Zhang et al., Integrated lithium niobate electro-optic modulators: when performance meets scalability, Optica 8, 652-

667 (2021). doi: 10.1364/OPTICA.415762
[3] C. Wang et al., Integrated lithium niobate electro-optic modulators operating at CMOS-compatible voltages, Nature 562, 101–104 (2018). doi: 10.1038/s41586-018-0551-y

Refs. [1-2] used a reference frequency of 1 GHz. Ref. 3 did not disclose the reference frequency, but its result (Fig. 2a) does not appear to correspond to a reference frequency of 2 GHz. If the authors would like to maintain consistency with previous works, they should not use a reference frequency of 2 GHz.

In addition, Ref. [1-3] are relatively old, and the EO response near DC has been further investigated in more recent works. In the rebuttal letter, the authors mention that "EO response near DC in lithium niobate devices can be affected by slow phenomena such as photorefractive effects, charge screening, thermal drift, and electrode resistance", and refer to these references:
[4] J. Holzgrafe et al., Relaxation of the electro-optic response in thin-film lithium niobate modulators, Optics Express 32, 3619–3631 (2024). doi: 10.1364/OE.507536
[5] R. Li et al., Low-Frequency Time-Domain Response of Thin-Film Lithium Niobate Electro-Optic Modulator, Photonics 13, 339 (2026). doi: 10.3390/photonics13040339

However, Ref. 4 states that "We consistently find across all devices that the EO response is nearly constant at high frequencies (measured at 100 kHz to 1 MHz)". This implies that a reference frequency of 1 MHz should be sufficient to avoid the influence of slow phenomena, which does not justify the choice of a 2 GHz reference frequency in this work.
Similarly, Ref. 5 states that "the study reveals that modulators with cladding exhibit a relaxation time of approximately 11.9 ms, showing positive DC drift and large drift. In contrast, modulators without cladding demonstrate a reduced relaxation time of about 88.6 μs with negative DC drift and small drift." This also does not justify the choice of a 2 GHz reference frequency.

Moreover, several recent works on TFLN modulators used reference frequencies much lower than 1 GHz, such as Refs. 6-7:
[6] Zhang, K., Sun, W., Chen, Y. et al. A power-efficient integrated lithium niobate electro-optic comb generator. Commun Phys 6, 17 (2023). https://doi.org/10.1038/s42005-023-01137-9
[7] Didier, P., Jain, P., Bertrand, M. et al. Thin film lithium niobate on sapphire for integrated mid-infrared modulator. Nat Commun 17, 3050 (2026). https://doi.org/10.1038/s41467-026-69880-5
Ref. 6 used a reference frequency of 10 MHz. Ref. 7 did not disclose the reference frequency, but the results (Fig. 4a-4b) indicate a low reference frequency.

Therefore, I am still not convinced that 2 GHz is an appropriate reference frequency for extracting the 3-dB bandwidth. The reference frequency should be carefully chosen for a fair comparison.

2. In Fig. 3d, the authors only measured the performance within 27~34 GHz. Can the performance below 27 GHz also be measured? Adding several data points between the reference frequency and 27 GHz is highly recommended.

3. Ref. 36 has been published as:
Didier, P., Jain, P., Bertrand, M. et al. Thin film lithium niobate on sapphire for integrated mid-infrared modulator. Nat Commun 17, 3050 (2026). https://doi.org/10.1038/s41467-026-69880-5
The reference should be updated.

(Remarks on code availability)

Reviewer #5

(Remarks to the Author)
I co-reviewed this manuscript with one of the reviewers who provided the listed reports. This is part of the Nature Communications initiative to facilitate training in peer review and to provide appropriate recognition for Early Career Researchers who co-review manuscripts.

(Remarks on code availability)

Version 2:

Reviewer comments:

Reviewer #4

(Remarks to the Author)
The authors have revised the manuscript to address my concerns. However, the revised manuscript still have some errors. For example, Figure 3(b) caption: "The measured reflection is below 25 dB from 2 – 40 GHz range indicating a well-matched impedance to 50 Ω." It should be -25 dB rather than "25 dB". Moreover, the S11 curve in Fig. 3(b) clearly contains regions above -25 dB, particularly in the 30-40 GHz range. The claim of "below -25 dB from 2 – 40 GHz range" is incorrect.
The authors should carefully check their manuscript to avoid such errors.

(Remarks on code availability)

Reviewer #5

(Remarks to the Author)
I co-reviewed this manuscript with one of the reviewers who provided the listed reports. This is part of the Nature Communications initiative to facilitate training in peer review and to provide appropriate recognition for Early Career Researchers who co-review manuscripts.

(Remarks on code availability)

Open Access This Peer Review File is licensed under a Creative Commons Attribution 4.0 International License, which permits use, sharing, adaptation, distribution and reproduction in any medium or format, as long as you give appropriate credit to the original author(s) and the source, provide a link to the Creative Commons license, and indicate if changes were made.
In cases where reviewers are anonymous, credit should be given to 'Anonymous Referee' and the source.
The images or other third party material in this Peer Review File are included in the article's Creative Commons license, unless indicated otherwise in a credit line to the material. If material is not included in the article's Creative Commons license and your intended use is not permitted by statutory regulation or exceeds the permitted use, you will need to obtain permission directly from the copyright holder.
To view a copy of this license, visit https://creativecommons.org/licenses/by/4.0/

Manuscript ID: NCOMMS-25-102205-T

"Suspended thin-film lithium niobate modulator for broadband mid-infrared light modulation and frequency comb generation"

Reply to Reviewers' Comments

We thank the Editor and the Reviewers for carefully reviewing our manuscript. We greatly appreciate the thoughtful and constructive comments provided during the review process. In the revised manuscript, we have addressed all comments and made corresponding revisions. All modifications in the manuscript are indicated in red text.

The Reviewers' comments are reproduced below in black, and our responses are provided in blue.

Reply to Reviewer #1

Reviewer #1 (Remarks to the Author):

Key results The authors demonstrate a suspended thin-film lithium-niobate platform enabling broadband MIR electro-optic modulation with low drive voltages ($V_\pi = 2.3 - 4.3V$) across 2.4–3.6 µm wavelengths. They achieve a 40 GHz EO bandwidth and report high-frequency V_π,MW of 4.5–6.5 V up to 35 GHz, representing substantial advances over prior MIR modulators. The work further showcases frequency-agile MIR EO comb generation with 0.8 THz 10 dB optical bandwidth and validates system-level performance in a free-space MIR communication link.

Validity The manuscript presents strong results and is, in my opinion, suitable for publication after minor revisions. The authors demonstrate noteworthy progress in MIR EO modulation. However, some points require clarification or correction to ensure accuracy and consistency.

We thank the Reviewer for the positive comment of our work. Below, we address the specific questions raised by the Reviewer:

1. Comment on the drawbacks of a suspended structure

TFLN is known to be sensitive to moisture and environmental factors, therefore usually it is cladded in real world applications. However, the fact that the structure is suspended, cladding it with oxide would not really be possible. It would be beneficial if the authors could comment on potential reliability issues such devices could face if this approach would be scaled up in the future or address approaches how this issue could be solved without hindering the performance metric benefits of such devices.

We thank the Reviewer for this insightful comment regarding the reliability and scalability of the suspended structure.

In this work, an air-suspended configuration is introduced to eliminate optical absorption from the buried oxide layer in the mid-infrared range while preserving strong optical confinement and high-speed microwave performance. As noted by the reviewer, thin-film lithium niobate devices operating in the mid-IR band may exhibit limited long-term stability under ambient conditions if left unpassivated. This degradation is primarily associated with surface adsorption of water and other airborne contaminants, which can increase optical loss at mid-IR wavelengths.

A promising strategy to address this issue is the use of plasma-enhanced atomic layer deposition (PE-ALD) passivation films, as recently demonstrated by D. Suk and co-workers [D. Suk et al., APL Mater. 13, 081102 (2025). doi: 10.1063/5.0275180]. Their study showed that ultrathin alumina and titanium dioxide coatings, with thicknesses on the order of tens of nanometers, can effectively suppress long-term loss degradation by serving as barriers against environmental moisture and contaminants. We anticipate that a similar approach can be readily applied to air-suspended thin-film lithium niobate platforms. Owing to the extremely thin coating thickness compared with the optical wavelength (about 1%), such passivation layers are expected to have negligible impact on optical confinement and microwave performance, while substantially improving environmental robustness. This approach therefore provides a practical path toward reliable mid-IR electro-optic modulators based on suspended thin-film lithium niobate.

To clarify this point, we have revised the manuscript as follows:
"The optical loss can be improved.... In addition, recent studies suggest that ultrathin plasma-enhanced atomic layer deposited passivation coatings [55] could potentially improve the environmental robustness and long-term stability of the mid-IR electro-optic modulators. A straightforward yet crucial next step..."
(Page 4)

2. Clarification of V_π versus V_π·L values

In several parts of the manuscript, the distinction between V_π and $V_\pi L$ is not consistently maintained, and in some cases the two are used interchangeably. This may create confusion for readers. For example, in the section:

"The corresponding half-wave voltage length product ($V_\pi L$) is 4.6 V cm at 2.4 µm and 8.6 V cm at 3.6 µm wavelength, respectively. The V_π scales near linearly with the optical wavelength λ...As the suspended optical waveguide supports guided mode over an ultrabroad bandwidth, we tested the V_π at the NIR wavelength of 1.55 µm to be 1.8 V..."

To improve clarity and enable a direct comparison, I recommend that the $V_\pi L$ value at 1.55 µm be

explicitly stated, just as is done for the MIR measurements.

Additionally, the manuscript uses $V_\pi L$ as a figure of merit without clearly specifying that the reported values correspond to a push–pull amplitude modulator, where the effective interaction length is twice the physical arm length. The conventional definition of $V_\pi L$ originates from phase modulators with a single-arm interaction length. If the authors indeed report the effective push–pull $V_\pi L$, this should be clearly stated to avoid ambiguity and ensure correct interpretation.

We thank the Reviewer for this insightful comment and appreciate the suggestion to clarify the distinction between V_π and $V_\pi L$.

Following the Reviewer's recommendation, we have revised the manuscript to explicitly distinguish between V_π and $V_\pi L$ throughout the text. In particular, we have now explicitly stated the corresponding $V_\pi L$ of 3.6 V cm at 1.55 µm to enable a direct comparison with the MIR measurements. We have also clarified that the reported $V_\pi L$ values correspond to a push–pull amplitude modulator configuration, where the effective interaction length is twice the physical arm length.

These clarifications have been incorporated in the main text and figure captions to ensure consistent terminology and unambiguous definition of the figure of merit.

To clarify this point, we have revised the manuscript as follows:
"As the suspended optical waveguide supports..., We test the $V_\pi L$ at the NIR wavelength of 1.55 µm to be 3.6 V cm, demonstrating our integrated EO.... Here, the reported $V_\pi L$ values are based on a Mach–Zehnder-interferometer amplitude modulator operated in a push–pull configuration. The extinction ratio..."
(Page 2)
"In addition, the suspended AM device is measured across more than an octave optical bandwidth with a half-wave voltage of 1.8 V at 1.55 μm, corresponding to a $V_\pi L$ of 3.6 V cm."
(Page 6, figure 2 - caption)

3. Incorrect referencing in Figures 2c and 3e

In both Figure 2c and Figure 3e, the numbering of the references in the plot does not match the references in Table 1 and the Bibliography. This must be corrected to avoid confusion and ensure that the comparisons presented in the figures can be accurately traced back to their corresponding sources. I recommend carefully rechecking all figure reference indices in the text and Table 1.

We thank the Reviewer for pointing out this issue and apologize for the inconsistency in the reference numbering.

Following the Reviewer's suggestion, we have carefully rechecked all reference indices in Figures 2c and 3e, as well as in Table 1 and the Bibliography. The incorrect numbering has been corrected in

the revised figures, and all references are now consistently matched across the figures, table, and reference list.

These corrections have been implemented in Figures 2c and 3e of the revised manuscript.

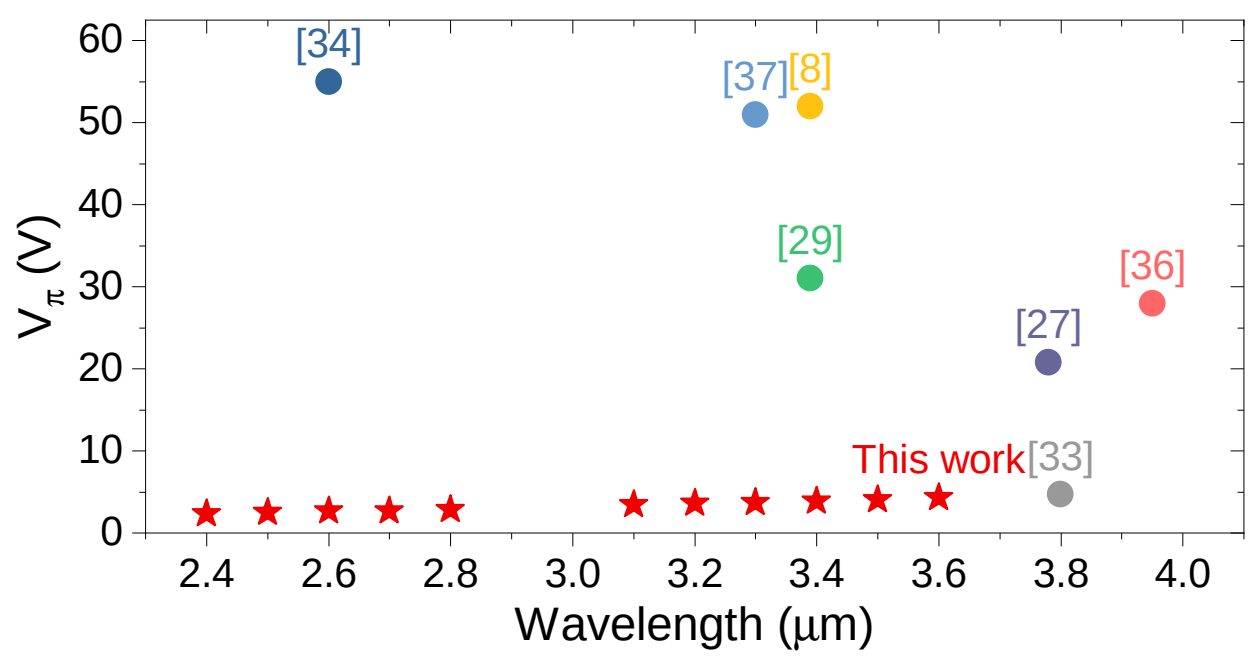

Figure R1: **The updated Fig. 2c in the revised manuscript.**

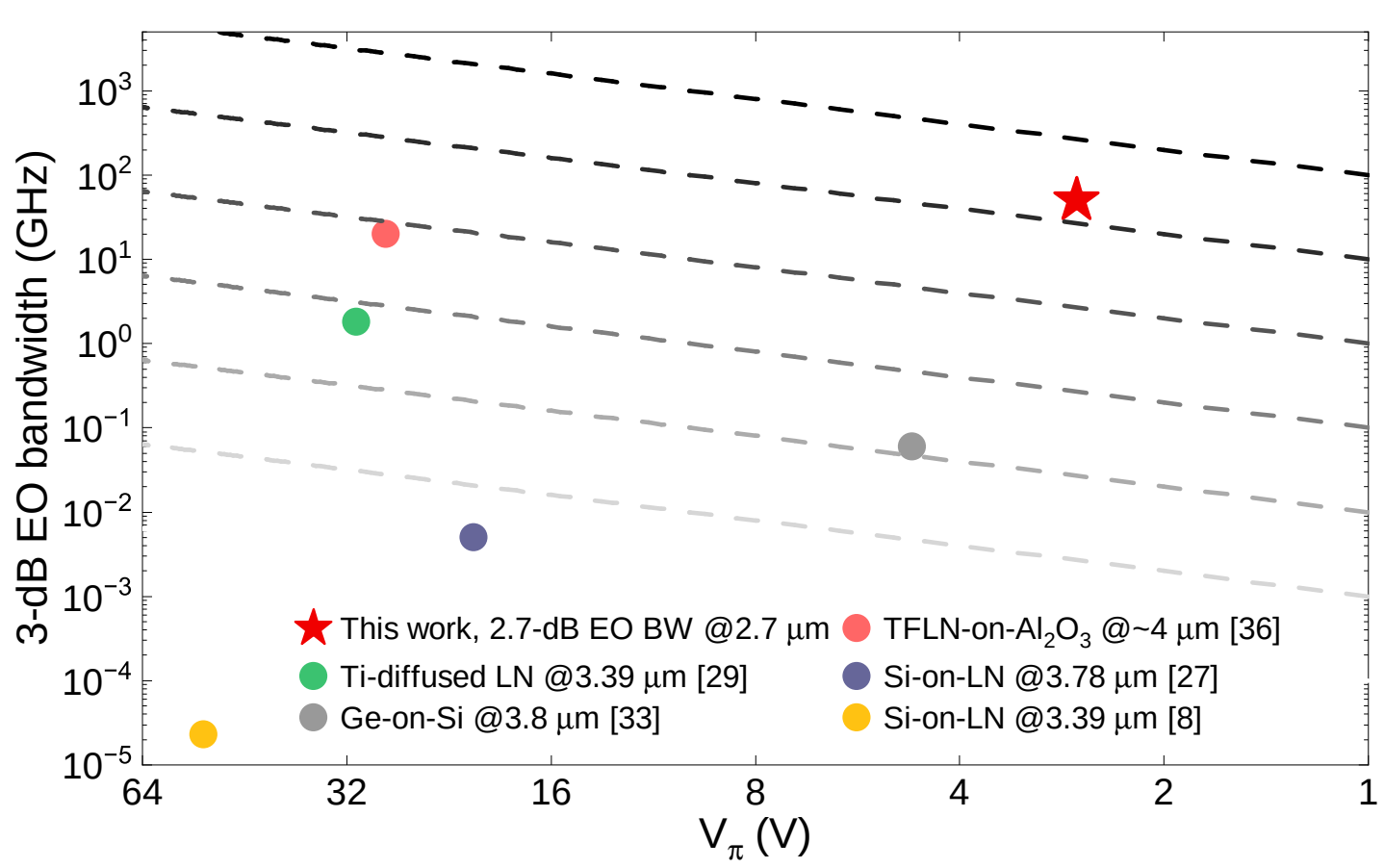

Figure R2: **The updated Fig. 3e in the revised manuscript.**

(Page 6, figure 2c) (Page 7, figure 3e)

4. Use of BW/V_π (GHz/V) as a figure of merit

The manuscript uses BW/V_π (GHz/V) as a figure of merit and compares it with prior modulators. This FOM is only valid in an oversimplified scenario as its validity relies on the assumption that the EO bandwidth scales inversely with interaction length under attenuation-limited, near velocity-matched conditions. Standard analytical formulations in the literature explicitly show that in general, linear length dependence for bandwidth cannot be directly assumed [1], [2], [3]. Since different works may be limited by different mechanisms (like RF attenuation, velocity mismatch, parasitics etc), cross-paper comparison using BW/V_π may not be physically equivalent, thus being

a somewhat meaningless way of comparison.

I recommend that the authors either
(i) clarify the physical regimes under which this FOM is meaningful and explicitly state the assumptions behind the comparison with other works, or
(ii) consider omitting this FOM from the text and figures as the manuscript already demonstrates superior performance relative to the literature.

[1] D. Zhu et al., "Integrated photonics on thin-film lithium niobate," Advances in Optics and Photonics, vol. 13, no. 2, p. 242, May 2021, doi: https://doi.org/10.1364/aop.411024
[2] J. Witzens, "High-Speed Silicon Photonics Modulators," Proceedings of the IEEE, vol. 106, no. 12, pp. 2158–2182, Dec. 2018, doi: https://doi.org/10.1109/jproc.2018.2877636
[3] G. Ghione, "Semiconductor Devices for High-Speed Optoelectronics," Oct. 2009,
doi: https://doi.org/10.1017/cbo9780511635595

We thank the Reviewer for this thoughtful and technically important comment regarding the use of BW/V_π as a figure of merit.

We agree that the BW/V_π metric is meaningful only under specific physical regimes, and that the electro-optic bandwidth does not universally scale inversely with interaction length when different limiting mechanisms (e.g., microwave attenuation, velocity mismatch, or parasitic loading) dominate device performance. Following the Reviewer's suggestion, we have revised the manuscript to explicitly clarify the assumptions under which this comparison is made.

In the revised text, we now state that the BW/V_π figure of merit is used as a practical system-level metric to compare devices operating in traveling-wave configurations with approximately velocity-matched microwave and optical modes and attenuation-limited response. We further clarify that this metric should be interpreted as an empirical performance indicator rather than a universal physical scaling law.

We also note that this figure of merit has been adopted in the literature for benchmarking high-speed electro-optic modulators, including prior thin-film lithium-niobate modulators reported in Nature:
[1] C. Wang et al., "Integrated lithium niobate electro-optic modulators operating at CMOS-compatible voltages," Nature, 562, 101-104, 2018. doi: 10.1038/s41586-018-0551-y

We have therefore added this citation in the revised manuscript and updated the discussion to ensure that the comparison is presented within the correct physical context.

These clarifications have been incorporated in the main text and figure captions to explicitly state the assumptions underlying the BW/V_π comparison, as follows:
"To the best knowledge...To note, another commonly used figure-of-merit (FOM) is the ratio be-

tween the 3-dB electro-optic bandwidth and the near-DC V_π (BW/V_π), which provides a practical performance metric for traveling-wave modulators operating near microwave–optical velocity matching, where the response is primarily limited by microwave attenuation [38]. This metric is used here as an empirical benchmark for comparison with prior works rather than as a universal physical scaling law. In our work, this FOM is...”
(Page 3)

Reply to Reviewer #2

Reviewer #2 (Remarks to the Author):
I co-reviewed this manuscript with one of the reviewers who provided the listed reports. This is part of the Nature Communications initiative to facilitate training in peer review and to provide appropriate recognition for Early Career Researchers who co-review manuscripts.

We thank Reviewer #2 and the co-reviewer for their careful evaluation of our manuscript and for their valuable time and effort.

Reply to Reviewer #3

Reviewer #3 (Remarks to the Author):
The work represents one of the first long wavelength electro optic modulators - if not the first - and as such has a respectable speed and voltage requirement when compared to alternative approaches at this wavelength. A comprehensive set of experiments give clarity on the new opportunities that may be foreseen.
The absense of good modulators in the mid infrared limits a wide range of use cases for integrated photonics, so this will be important for sensing and free space communications research. The work is carefully specified for mid infrared where there is limited prior art. Therefore the work is self consistent. The methods are sound. The only unaddressed question would be why the performance in mid infrared is not as good as near infrared in terms of bandwidth and efficiency. It would be of interest to know whether the authors has insight on this.

Reviewer #3 (Remarks on code availability):
no comment

We thank Reviewer #3 for the positive assessment of our work and for recognizing the potential impact of mid-infrared electro-optic modulators for sensing and free-space communication applica-

tions.

EO phase accumulation is expressed using

$$\Delta\phi = \Delta\beta L, \qquad \Delta\beta \propto n_e^3 r_{33} \Gamma \frac{2\pi}{\lambda}$$

where λ is the optical wavelength, n_e is the extraordinary refractive index, r_{33} is the electro-optic coefficient, and Γ is the microwave–optical mode overlap factor.

Therefore, the modulation efficiency decreases at longer wavelengths due to two primary factors. First, the larger optical mode size reduces the microwave–optical mode overlap. Second, the smaller optical wave vector ($k = 2\pi/\lambda$, unit: rad/m) leads to slower phase accumulation (Extended Data Fig. 1b).
The electro-optic phase shift can be quantitatively described using the relation adopted in Methods Section 2.2:

$$\Delta\beta_{\mathrm{TE}} = \frac{2n_e\,\Delta n_e}{2\beta_{\mathrm{TE}}} k_0^2 = -\frac{n_e^4 r_{33} \Gamma_{\mathrm{mo}}}{2\beta_{\mathrm{TE}}} k_0^2, \qquad V_\pi \cdot L = \frac{\pi}{\Delta\beta_{\mathrm{TE}}} \times 100\ (\mathrm{V \cdot cm})$$

To clarify this point, we have revised the discussion in Methods Section 2.2 (Page 11) to explicitly describe the wavelength scaling of modulation efficiency. The updated text now reads:
"Measured DC V_π values... We note that a higher V_π is expected at longer optical wavelengths due to both the reduced optical wave vector ($k = 2\pi/\lambda$), which slows phase accumulation per unit length, and the increased optical mode size, which decreases the microwave–optical mode overlap (Extended Data Fig. 1b)."
(Page 11)

In addition, we have added a brief clarification in the main text where the near-infrared $V_\pi L$ value is discussed to highlight this expected scaling behavior. The added sentence reads:
"As the suspended optical waveguide... The slightly higher $V_\pi L$ observed at longer wavelengths is consistent with the expected wavelength scaling of electro-optic phase accumulation, where reduced optical wave vector and increased optical mode size lead to lower modulation efficiency. The extinction ratio..."
(Page 2)

Reply to Reviewer #4

Reviewer #4 (Remarks to the Author):

This manuscript reports a suspended thin-film lithium niobate (TFLN) electro-optic platform for

mid-infrared (MIR) applications, demonstrating low half-wave voltage, broadband operation, and high electro-optic (EO) bandwidth. The authors further present an MIR EO frequency comb and a proof-of-concept free-space optical communication link. The work addresses an important challenge in MIR photonics and presents a promising device platform combining low drive voltage and high-speed operation. The manuscript is also well written. I recommend its publication after some revisions. My detailed comments are listed below.

We thank Reviewer #4 for the positive assessment of our work and for recognizing the importance of developing electro-optic modulators for mid-infrared applications. We also appreciate the Reviewer's constructive suggestions and have carefully revised the manuscript to address the points raised below.

1. While the authors claim "low-loss EO modulation", this is not sufficiently supported by the experimental results. For example, the measured active propagation loss at 3.6 µm is as high as 10.5 dB/cm. This result significantly weakens the claim of "low-loss EO modulation".

We thank the Reviewer for this important comment regarding the statement of "low-loss EO modulation".

We agree that the measured propagation loss of 10.5 dB/cm at 3.6 µm is significant and should be carefully interpreted. In our device, this loss includes contributions from both the passive waveguide and the metal electrodes used for electro-optic modulation.

Our intention was to emphasize the relatively low passive waveguide loss enabled by the suspended platform, rather than to imply that the total device loss in the active section is low. As shown in the table, our passive loss is comparable to other work.

To avoid confusion and prevent overstating the current device performance, we have removed the phrase "low-loss" from the manuscript. The revised text now reads:
"In addition, traditional silicon-based ....Therefore, a compact, high-speed and efficient EO modulator in the MIR is still missing."
(Page 2)
"Such systems would not .... The ability to combine efficient electro-optic modulation, broadband MIR transparency, and scalable chip-level integration marks an important step toward fully reconfigurable, multifunctional MIR photonic systems."
(Page 4)

Regarding the loss in the active modulation section, as stated in our insertion-loss analysis, the exact origin of the additional attenuation is still under investigation, since the simulated metal-induced losses are only 0.2 dB/cm and 4.8 dB/cm at 2.4 µm and 3.6 µm, respectively, which do not fully account for the measured values. One possible explanation is that high-energy electron

exposure during electron-beam lithography of the nearby metal electrodes may introduce excess free carriers or material damage near the waveguide surface, leading to increased free-carrier absorption that becomes more pronounced at longer wavelengths. This excess loss may be mitigated in future devices through optimized annealing processes and photolithography-based electrode fabrication. We have also revised the manuscript to use more precise language distinguishing passive waveguide loss from total device loss.

2. Characterizing the bandwidth of a modulator typically requires a high-speed photodetector (PD). However, this approach is not adopted in this work. I guess the reason is the lack of commercially available high-speed PDs at these wavelengths. If so, the authors should clarify this point. On the other hand, commercial high-speed PDs are available around 2.6 µm. The authors may consider characterizing the modulator's bandwidth at 2.6 µm using a commercial high-speed PD. In addition, the 3 dB bandwidth is referenced to the performance at 2 GHz, which is not commonly seen in the literature. It is recommended to define the bandwidth relative to the near-DC response (e.g., kHz or MHz).

We thank the Reviewer for this constructive comment regarding the bandwidth characterization methodology.

We agree that using a high-speed photodetector is a standard and direct approach for measuring the electro-optic bandwidth of modulators when suitable detectors are available. However, in the mid-infrared wavelength range studied here, especially beyond 3 µm, commercial high-speed photodetectors with sufficient bandwidth are not readily available.

In such cases, an equivalent and widely adopted method is to extract the electro-optic response from the optical modulation sidebands measured in the spectral domain. This approach has also been used extensively in near-infrared TFLN modulators when detector bandwidth becomes limiting. For example, lithium niobate EO modulators have employed optical-spectrum-based measurements at high frequencies beyond 40 GHz detector limits (figure 4a in [C. Wang et al, Integrated lithium niobate electro-optic modulators operating at CMOS-compatible voltages, Nature 562, 101–104 (2018). 10.1038/s41586-018-0551-y]). A detailed description of this method can be found in X. Chen et al., Characterization of Electro-Optic Bandwidth of Ultra-High Speed Modulators, OFC 2017, paper Tu2H.7. doi: 10.1364/OFC.2017.Tu2H.7).

In our work, we apply the same principle using an FTIR spectrometer, which serves as the mid-infrared analogue of an optical spectrum analyzer. Specifically, we measure the modulation sideband power under RF drive, from which the microwave V_π and corresponding EO frequency response are extracted. This method directly measures modulation efficiency and avoids reliance on unavailable high-speed mid-IR photodetectors.

Regarding the choice of offset reference frequency, we note that in TFLN modulators the EO roll-off is often defined relative to a low but non-zero reference frequency, typically 1–2 GHz, rather than true DC. This approach has been used in several prior works on high-speed thin-film lithium niobate modulators, including

[1] P. Kharel et al., Breaking voltage–bandwidth limits in integrated lithium niobate modulators using micro-structured electrodes, Optica 8, 357-363 (2021). doi: 10.1364/OPTICA.416155

[2] M. Zhang et al., Integrated lithium niobate electro-optic modulators: when performance meets scalability, optica 8, 652-667 (2021). doi: 10.1364/OPTICA.415762

[3] C. Wang et al., Integrated lithium niobate electro-optic modulators operating at CMOS-compatible voltages, Nature 562, 101–104 (2018). doi: 10.1038/s41586-018-0551-y

The reason is that EO response near DC in lithium niobate devices can be affected by slow phenomena such as photorefractive effects, charge screening, thermal drift, and electrode resistance, which may lead to over- or under-estimation of the effective V_π. Therefore, referencing the roll-off to 1–2 GHz generally provides a more reliable and comparable metric for assessing true RF modulation performance. Relevant discussions of low-frequency response behavior and slow dynamic effects in lithium niobate electro-optic devices can be found in:

[1] J. Holzgrafe et al., Relaxation of the electro-optic response in thin-film lithium niobate modulators, Optics Express 32, 3619–3631 (2024). doi: 10.1364/OE.507536

[2] R. Li et al., Low-Frequency Time-Domain Response of Thin-Film Lithium Niobate Electro-Optic Modulator, Photonics 13, 339 (2026). doi: 10.3390/photonics13040339

We have revised the manuscript to clarify both the FTIR sideband measurement method and the 2 GHz reference frequency for EO bandwidth extraction as follows:

"The MW V_π is extracted ....High-speed photodetectors operating beyond 3 µm with bandwidths exceeding tens of gigahertz are currently limited in availability, and therefore the electro-optic response is characterized using optical modulation sidebands measured in the spectral domain [38,39]. Figure 3d plots the measured MW V_π .... The electro-optic response is referenced to a non-zero MW frequency of 2 GHz rather than the DC [38,40,41] to minimize the influence of slow device dynamics at low frequencies, such as charge screening and photorefractive effects, which can affect the measured modulation response [42,43]. The EO response decreases by 2.7 dB at a MW frequency of 40 GHz, which is the frequency limit of our signal generator. By fitting the measured response and referencing the 2 GHz response, the extracted 3-dB EO bandwidth is estimated to be 50 GHz (dashed line). To the best knowledge, ..."

(Page 3)

We have also revised the corresponding caption to align with the main text:

"(d) Microwave V_π and the corresponding electro-optic response, referenced to a non-zero frequency of 2 GHz. Microwave V_π values ... measured S parameters in (a). The electro-optic response scales

inversely with the square of the microwave V_π and decreases by 2.7 dB at 40 GHz, which is the frequency limit of our signal generator, while the extracted 3-dB EO bandwidth is 50 GHz (dashed line).
(Page 7)

3. The authors provided several design parameters, such as the metal gap and waveguide height. However, details regarding the air holes are missing. For example, the hole size, hole pitch, and distance from the waveguide core are not provided. These parameters should also be included.

We thank the Reviewer for this helpful suggestion.

We agree that the geometric parameters of the air-hole structure are important for reproducibility and clarity. Following the Reviewer's recommendation, we have added the corresponding design details, including the hole diameter, hole pitch, and the distance between the air holes and the waveguide core, in the revised manuscript.

To address this point, we have revised the manuscript as follows:
"Air holes are then defined .... The periodic array of air holes, used to suspend the waveguide through wet undercut etching, has a pitch of 13.5 µm, dimensions of 3.5 µm × 1 µm, and a center-to-waveguide distance of 5.75 µm. The air holes are placed in between ..."
(Page 2)

4. Figure 4(b): While a 0.8-THz-span MIR EO comb with more than 30 lines is interesting, the results shown in Fig. 4(b) appear preliminary, particularly given the large power variation among the comb lines. It is not clear what practical applications can be enabled by this MIR EO comb.

We thank the Reviewer for this insightful comment regarding the spectral flatness and potential applications of the demonstrated MIR EO comb.

We agree that the current comb spectrum exhibits noticeable power variation among the comb lines. We note, however, that a perfectly flat spectral envelope may not be strictly required for many practical applications of EO combs, such as molecular spectroscopy, chemical sensing, and wavelength calibration. In these contexts, key attributes often include phase coherence, stable line spacing, mutual coherence among comb teeth, and sufficient optical power in the relevant spectral regions, rather than uniform amplitude across the entire comb.

The demonstrated comb is generated using a single phase modulator driven by a single-frequency microwave signal, with the spectral envelope primarily determined by the Bessel-function distribution associated with the modulation index. As shown in Fig. 4, we focus here on an EO comb generated using a phase-modulation architecture. Compared with intensity-modulation-based approaches, a phase modulator in the present double-pass configuration can, under similar drive

conditions, provide a broader optical comb bandwidth. In addition, phase modulators can support a range of functionalities beyond comb generation, including frequency shifting, chirping, arbitrary waveform synthesis, time-lens operation, serrodyne modulation, and spectro-temporal signal processing. These considerations suggest that the demonstrated phase-modulation platform may offer additional flexibility for integrated MIR photonics."

We would also like to note that spectral flatness can be improved using established EO comb synthesis techniques. In particular, incorporating an intensity modulator before the phase modulator can shape the temporal waveform and lead to a flatter comb envelope after phase modulation.

Such approaches enable deterministic control of the comb envelope and have been demonstrated in integrated electro-optic comb systems in the near IR regime:

[1] M. Yu et al., "Integrated femtosecond pulse generator on thin-film lithium niobate," Nature, vol. 612, p. 252-258, 2022, doi: 10.1038/s41586-022-05345-1.

[2] C-H. Lee et al., "Ultra-broadband Flat-Top Electro-Optic Frequency Combs on a Lithium Niobate Platform," in CLEO 2025, Technical Digest Series (Optica Publishing Group, 2025), paper SS164_3, doi: 10.1364/CLEO_SI.2025.SS164_3

[3] X. Ren et al., "Few-cycle Pulse Generation on the Thin-film Lithium Niobate Platform," in CLEO 2025, Technical Digest Series (Optica Publishing Group, 2025), paper SS150_3, doi: 10.1364/CLEO_SI.2025.SS150_3.

[4] R. Cheng et al., "Single-drive electro-optic frequency comb source on a photonic-wire-bonded thin-film lithium niobate platform," Optics Letters, vol. 49, p. 3504-3507, 2024, doi: 10.1364/OL.527659.

[5] R. wu et al., "Generation of very flat optical frequency combs from continuous-wave lasers using cascaded intensity and phase modulators driven by tailored radio frequency waveforms," Optics Letters, vol. 35, p. 3234-3236, 2010, doi: 10.1364/OL.35.003234.

Therefore, we view the presently observed non-flat spectrum as a characteristic of the simple single-stage modulation architecture rather than a fundamental limitation of the platform. With integrated cascaded modulators and waveform engineering, substantially flatter and broader MIR comb spectra should be achievable in future implementations.

We have added a brief discussion in the revised manuscript to clarify this point and to outline possible approaches toward achieving flatter comb spectra, which we are currently investigating.

To address this point, we have revised the manuscript as follows:

"The total modulation index (β) ....The current comb spectrum is generated using a phase-modulation-only configuration, and the spectral envelope is therefore not actively flattened. Improved spectral flatness can be achieved by incorporating amplitude modulation or time-lens techniques prior to phase modulation, as demonstrated in prior work and currently under investigation [35, 44-47]. This is the first demonstration ..."

(Page 3)

5. Figure 1(f): The claimed waveguide width (w0) of 4 m is not consistent with the two scale bars in this figure. The authors should double-check the scale bars in all figures.

We thank the Reviewer for carefully raising this point.

The figure is correct, but we agree that the presentation could lead to confusion. Specifically, the SEM image and optical mode profile shown in Fig. 1(f) corresponds to the cleaved facet region used for edge coupling, where the waveguide width is 2 µm. Along the propagation direction, the waveguide is gradually expanded to a width of 4 µm in the modulation section, which corresponds to the parameter w_0 referenced in the manuscript.

To clarify this point, we have revised the description to explicitly distinguish between the facet width and the modulation-region width.
"The cross-section of ...profile at 3 μm. Both the SEM image and the simulated optical mode profile shown in Fig. 1f correspond to the waveguide at the cleaved facet region used for edge coupling, where the waveguide width is approximately 2 µm. After input coupling, the waveguide width is gradually tapered to the designed width of 4 µm used in the active modulation section."
(Page 2)

6. Figure 5(a): Please check the use of "OSC" in the figure and "DSO" in the caption for consistency.

We thank the Reviewer for pointing out this inconsistency.

We have checked the labeling in Fig. 5(a) and the corresponding caption, and corrected the label to ensure consistency. Specifically, the abbreviation has been unified to "DSO" (digital storage oscilloscope) throughout the figure and caption.
(Page 8, Fig. 5(a))

7. Table 1: Please check whether "PAM8@1.5 Gbaud" is correct.

We thank the Reviewer for carefully checking this detail.

We confirm that the entry "PAM8@1.5 Gbaud" in Table 1 was incorrect. The correct symbol rate is 0.5 Gbaud. We have revised Table 1 accordingly to reflect the accurate modulation format.
(Page 9, Table 1)

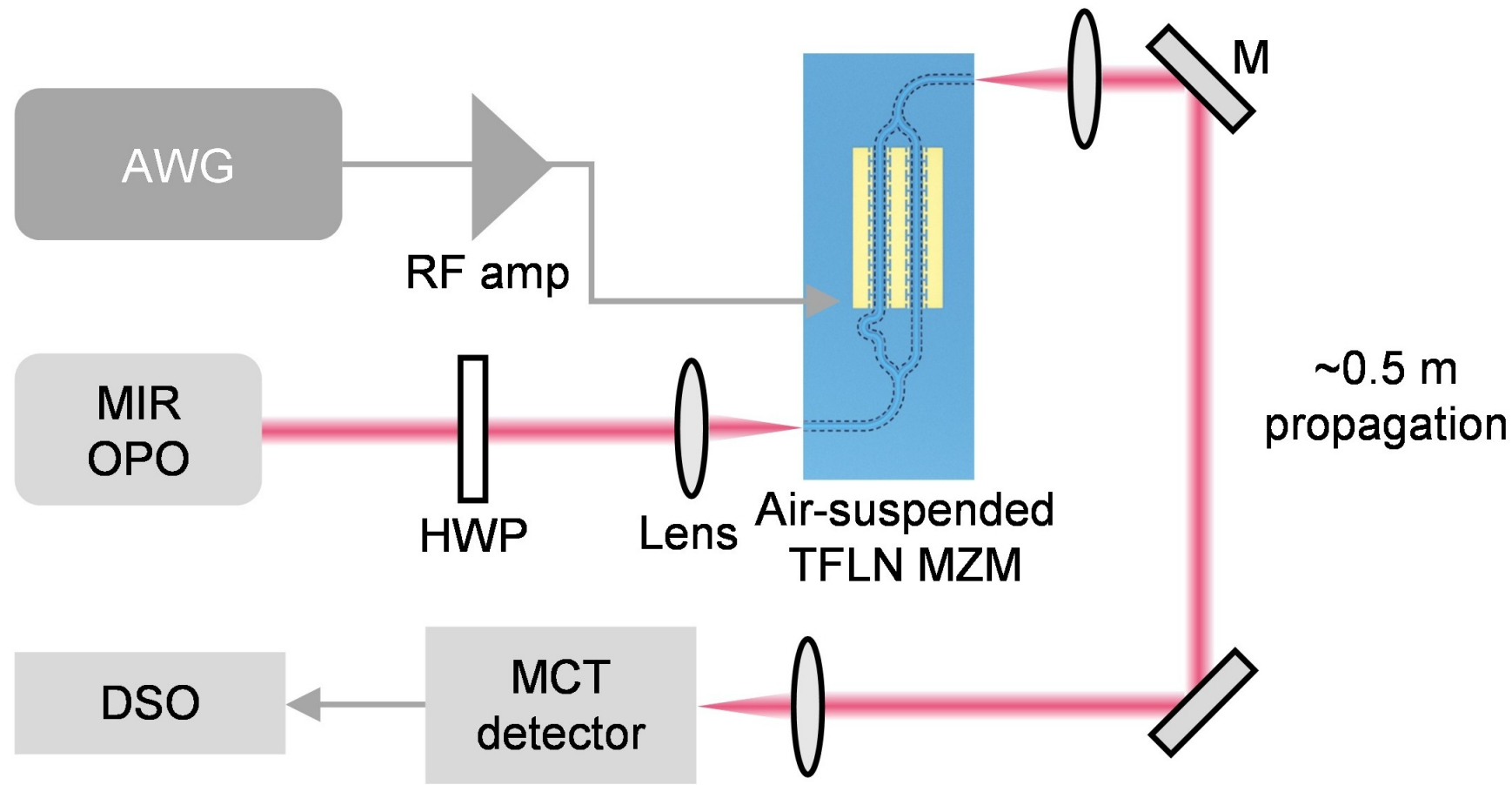

Figure R3: **The updated Fig. 5a in the revised manuscript.**

Reply to Reviewer #5

Reviewer #2 (Remarks to the Author):
I co-reviewed this manuscript with one of the reviewers who provided the listed reports. This is part of the Nature Communications initiative to facilitate training in peer review and to provide appropriate recognition for Early Career Researchers who co-review manuscripts.

We thank Reviewer #5 and the co-reviewer for careful assessment of our manuscript and for their constructive feedback.
We hope that the revisions and clarifications provided in this response and the revised manuscript adequately address the reviewers' comments.
We sincerely thank the Editor and the Reviewers for their time and consideration.

Manuscript **NCOMMS-25-102205A**
"Suspended thin-film lithium niobate modulator for broadband mid-infrared light modulation and frequency comb generation"
Reply to Reviewers' Comments:

We thank the editor, reviewers, and co-reviewers for their careful evaluation of our manuscript. We have revised the manuscript accordingly, and the reviewer comments are reproduced below in black, followed by our responses in blue.

Reply to Reviewer #1

Reviewer #1 (Remarks to the Author):

Both the main reviewer and the co-reviewer reviewed the authors' response and we think that our questions and concerns were properly addressed. We think that the paper is fit to be published.

We sincerely thank the reviewer and co-reviewer for their careful evaluation and positive assessment of our revised manuscript. We greatly appreciate their time and constructive feedback throughout the review process.

Reply to Reviewer #2

Reviewer #2 (Remarks to the Author):

I co-reviewed this manuscript with one of the reviewers who provided the listed reports. This is part of the Nature Communications initiative to facilitate training in peer review and to provide appropriate recognition for Early Career Researchers who co-review manuscripts.

We thank the co-reviewer for their time and contribution to the evaluation of our manuscript.

Reply to Reviewer #4:

Reviewer #4 (Remarks to the Author):

The authors have addressed most of my concerns, but some new concerns have arisen after reading the revised manuscript and the rebuttal letter. Before publication, the authors should address the following concerns.

1. In the rebuttal letter, the authors claim that “the EO roll-off is often defined relative to a low but non-zero reference frequency, typically 1-2 GHz”, and refer to these references:
[1] P. Kharel et al., Breaking voltage–bandwidth limits in integrated lithium niobate modulators using micro-structured electrodes, Optica 8, 357-363 (2021). doi: 10.1364/OPTICA.416155
[2] M. Zhang et al., Integrated lithium niobate electro-optic modulators: when performance meets scalability, Optica 8, 652-667 (2021). doi: 10.1364/OPTICA.415762
[3] C. Wang et al., Integrated lithium niobate electro-optic modulators operating at CMOS-compatible voltages, Nature 562, 101–104 (2018). doi: 10.1038/s41586-018-0551-y

Refs. [1-2] used a reference frequency of 1 GHz. Ref. 3 did not disclose the reference frequency, but its result (Fig. 2a) does not appear to correspond to a reference frequency of 2 GHz. If the authors would like to maintain consistency with previous works, they should not use a reference frequency of 2 GHz.

In addition, Ref. [1-3] are relatively old, and the EO response near DC has been further investigated in more recent works. In the rebuttal letter, the authors mention that “EO response near DC in lithium niobate devices can be affected by slow phenomena such as photorefractive effects, charge screening, thermal drift, and electrode resistance”, and refer to these references:
[4] J. Holzgrafe et al., Relaxation of the electro-optic response in thin-film lithium niobate modulators, Optics Express 32, 3619–3631 (2024). doi: 10.1364/OE.507536
[5] R. Li et al., Low-Frequency Time-Domain Response of Thin-Film Lithium Niobate Electro-Optic Modulator, Photonics 13, 339 (2026). doi: 10.3390/photonics13040339

However, Ref. 4 states that “We consistently find across all devices that the EO response is nearly constant at high frequencies (measured at 100 kHz to 1 MHz)”. This implies that a reference frequency of 1 MHz should be sufficient to avoid the influence of slow phenomena, which does not justify the choice of a 2 GHz reference frequency in this work.

Similarly, Ref. 5 states that “the study reveals that modulators with cladding exhibit a relaxation time of approximately 11.9 ms, showing positive DC drift and large drift. In contrast, modulators without cladding demonstrate a reduced relaxation time of about 88.6 µs with negative DC drift and small drift.” This also does not justify the choice of a 2 GHz reference frequency.

Moreover, several recent works on TFLN modulators used reference frequencies much lower than 1 GHz, such as Refs. 6-7:
[6] Zhang, K., Sun, W., Chen, Y. et al. A power-efficient integrated lithium niobate electro-optic comb generator. Commun Phys 6, 17 (2023). https://doi.org/10.1038/s42005-023-01137-9

[7] Didier, P., Jain, P., Bertrand, M. et al. Thin film lithium niobate on sapphire for integrated mid-infrared modulator. Nat Commun 17, 3050 (2026). https://doi.org/10.1038/s41467-026-69880-5
Ref. 6 used a reference frequency of 10 MHz. Ref. 7 did not disclose the reference frequency, but the results (Fig. 4a-4b) indicate a low reference frequency.

Therefore, I am still not convinced that 2 GHz is an appropriate reference frequency for extracting the 3-dB bandwidth. The reference frequency should be carefully chosen for a fair comparison.

We thank the reviewer for the detailed comments regarding the reference frequency used by the EO modulator community. We apologize for not being clear in our rebuttal letter and the main texts.

The reference frequency can be chosen at or near DC, but may also be taken at a higher frequency (e.g., 1 GHz or other values). Let's first explain the rationale behind each choice.

First of all, the EO response EO S_{21} is often used to reflect the MW Vπ (Vπ, MW) roll off as compared to the reference low-frequency (Vπ, LF), using the equation below:

$$V_{\pi,\mathrm{RF}} = V_{\pi,\mathrm{LF}} \times 10^{-EO\,S_{21}/20}$$

Ultimately the microwave Vπ value determines the modulation efficiency. If one did not directly measure the Vπ value across different frequencies, one can often use the EO response together with the Vπ,LF at the reference frequency to predict the MW Vπ value (based on the equation). In our case, we directly measure the MW Vπ value (from 27-34 GHz range by FTIR, and from 100 kHz to 0.4 GHz range, see added data in fig. 3b).
So if the EO response is nearly smooth from the chosen reference frequency to MW frequency range (no large oscillation), the reflection of Vπ roll off is very clear. However it is often not the case due to the following reasons: 1) one reason is the slow drift due to photorefractive effect, charge screening typically below kHz - MHz range, as reviewer also pointed out in the references; 2) Another reason is the impedance mismatch between the characteristic impedance of the transmission line Z_0 and the terminal load impedance Z_L. The reflection coefficient can be either negative or positive (a π phase difference) depending on the Z_0 - Z_L is positive or negative. The reflected MW at the termination will interfere with the co-propagating MW field and create a standing wave pattern, which induces a peak or rapid roll-off, and oscillation in the EO responses. This is why in some papers, one can see EO response is larger than 0 dB (we give a few examples later in the letter).

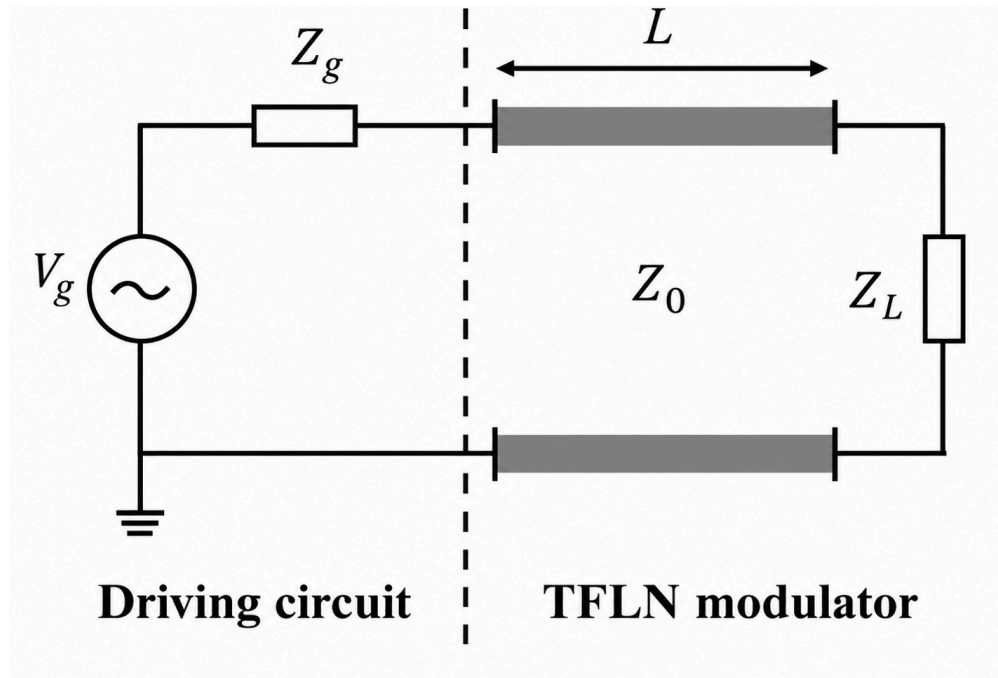

Fig S1 Equivalent circuit for the travelling-wave TFLN modulator with terminal. Z_g: source impedance (50 ohm), Z_0 transmission line impedance (by design), and Z_L is the terminal load impedance (user defined, often an off-chip termination connected to the GSG probe)

To give a better visualization, we simulated the EO response with different Z_L in Fig. S2a, reference to the DC frequency.

The E-O responses in Fig. S2a reveal that a terminal resistance ZL lower than the characteristic impedance Z_0 of coplanar transmission line results in an initial peak in the EO response, thus larger EO-bandwidth, explained by the negative reflection coefficient ($Z_L < Z_0$), whereas for $Z_L > Z_0$, the EO responses exhibits a sharp drop-off at low frequencies (also see equations below). The sensitivity of the 3-dB EO response bandwidth is largely due to the phase of the reflected coefficient where the DC frequency response is at the rising or decreasing slope of the oscillation induced by this impedance mismatch.

One important thing to note is that the increased EO bandwidth is achieved at a cost of higher DC Vπ, as seen in the Vπ simulation under the same conditions in Fig. S2b. When Z_L is less than Z_0, the DC Vπ value is also higher. As one can see the MW Vπ remains very similar between the three cases. From the Vπ perspective, the Z_L = 60 ohm case might be better due to a lower Vπ at DC frequency despite in the EO response it shows a rapid roll-off. In other words, EO response is from a relative perspective.

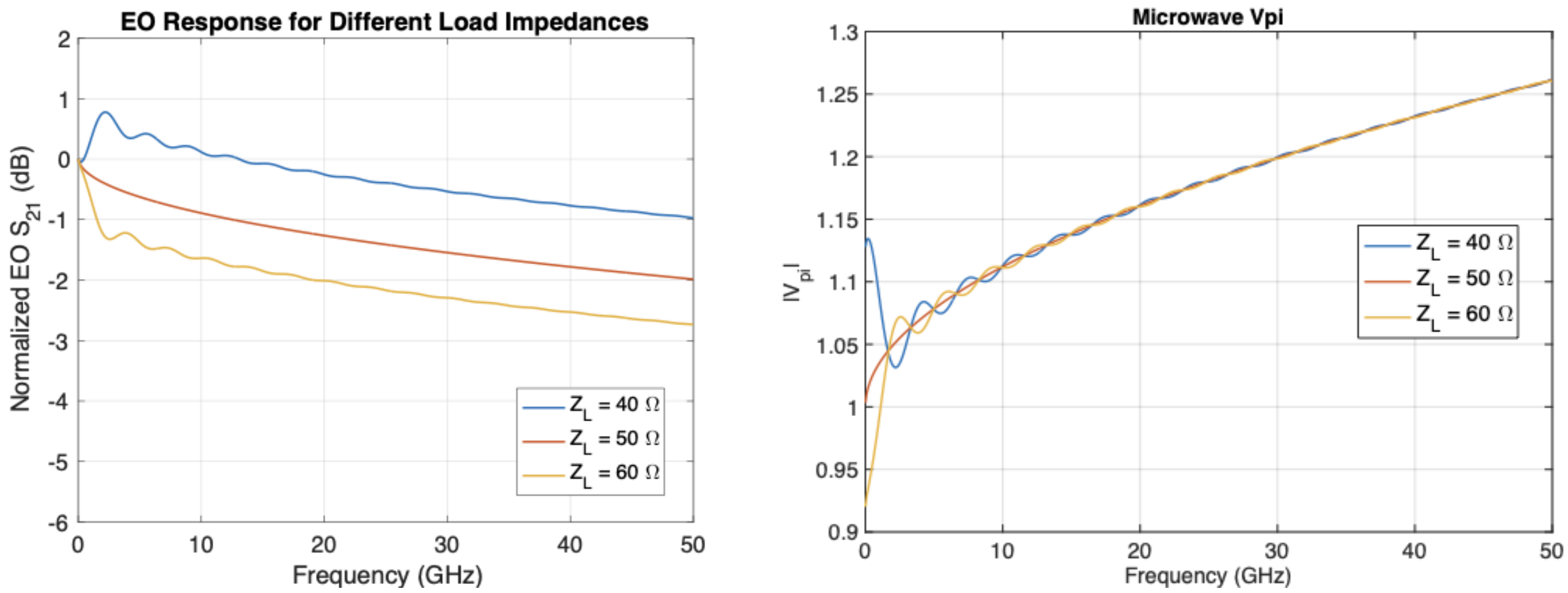

Fig. S2 (a) Simulated EO response from DC to 50 GHz, at three different load resistance (Z_L = 40, 50 (perfectly matched) and 60 ohm). The 3-dB EO bandwidth (when ref to the DC frequency) can be tuned largely by just termination impedance alone. (b) half-wave voltage Vπ, normalized to 1 V at perfect impedance matched condition. As one can see, the increased 3-dB EO bandwidth (Z_L = 40 ohm) leads to an increase of Vπ at DC frequency as well. Regardless of the loads, the MW Vπ at higher frequencies remains similar.

The EO response function can be modelled using the following equations

$$m(\omega) = 2\left|\frac{Z_{in}}{Z_{in}+Z_L}\right|\left|\frac{(Z_L+Z_0)F_+ + (Z_L-Z_0)F_-}{(Z_L+Z_0)e^{\gamma_m L} + (Z_L-Z_0)e^{-\gamma_m L}}\right|$$

$$F_\pm = \left(1-e^{\pm\gamma_m L - j\frac{\omega}{c_0}L}\right)/\left(\pm\gamma_m L - j\frac{\omega}{c_0}n_0 L\right) \qquad Z_{in} = Z_0\frac{Z_L + Z_0 tanh(\gamma_m L)}{Z_0 + Z_L tanh(\gamma_m L)} \qquad \gamma_m = \alpha_m + j\frac{\omega}{c_0}n_m$$

Considering all three factors mentioned above, the frequency response of an EO modulator follows [G. Ghione, "Chapter 6: Modulators," in *Semiconductor Devices for High-Speed Optoelectronics* (Cambridge University, 2009).]

where
w is the microwave frequency,
Zin is the input impedance of the transmission line,
F_{+-} accounts for the forward/backward propagating waves,
y_m is the complex microwave propagation constant (n_m being the microwave effective index, α_m being the loss rate),
n_0 is the optical group index,
L is the modulator length, and
c is the speed of light in free space.
We can also simulate the average voltage on the modulator using equations in ref [Appl. Opt. 26, 1696-1700 (1987)]:

1. Average microwave voltage:

$$V_{\mathrm{avg}}(\omega_m) = \frac{1}{L}\int_0^L \frac{V_g(1+\rho_1)e^{i\beta_o L}\left(e^{(\gamma_m - i\beta_o)x} + \rho_2 e^{-(\gamma_m + i\beta_o)x}\right)}{2(e^{\gamma_m L} + \rho_1\rho_2 e^{-\gamma_m L})}\, dx$$

2. Reflection coefficient:

$$\rho_1 = \frac{Z_0 - Z_g}{Z_0 + Z_g}, \qquad \rho_2 = \frac{Z_L - Z_0}{Z_L + Z_0}$$

3. Definitions:

$$\beta_o = \frac{\omega}{c_0} n_o$$

where $y_m - i\beta_o$ and $y_m + i\beta_o$ represent the wave-vector walk-off between the optical wave and the transmitted/reflected microwave signals, respectively, while ρ_1 and ρ_2 denote the input and output reflection coefficients of the microwave transmission line.

Therefore, the overall roll-off of Vπ vs frequency is revealed when one references a non-DC frequency when there is an impedance mismatch. As shown in Fig.S3 (also Fig. S2a), between all cases, if one picks the reference point (indicated by the arrow), one can recover a smooth roll-off profile which reflects the microwave index matching and attenuation achieved, which both are critical to the MW Vπ value and modulation efficiency.

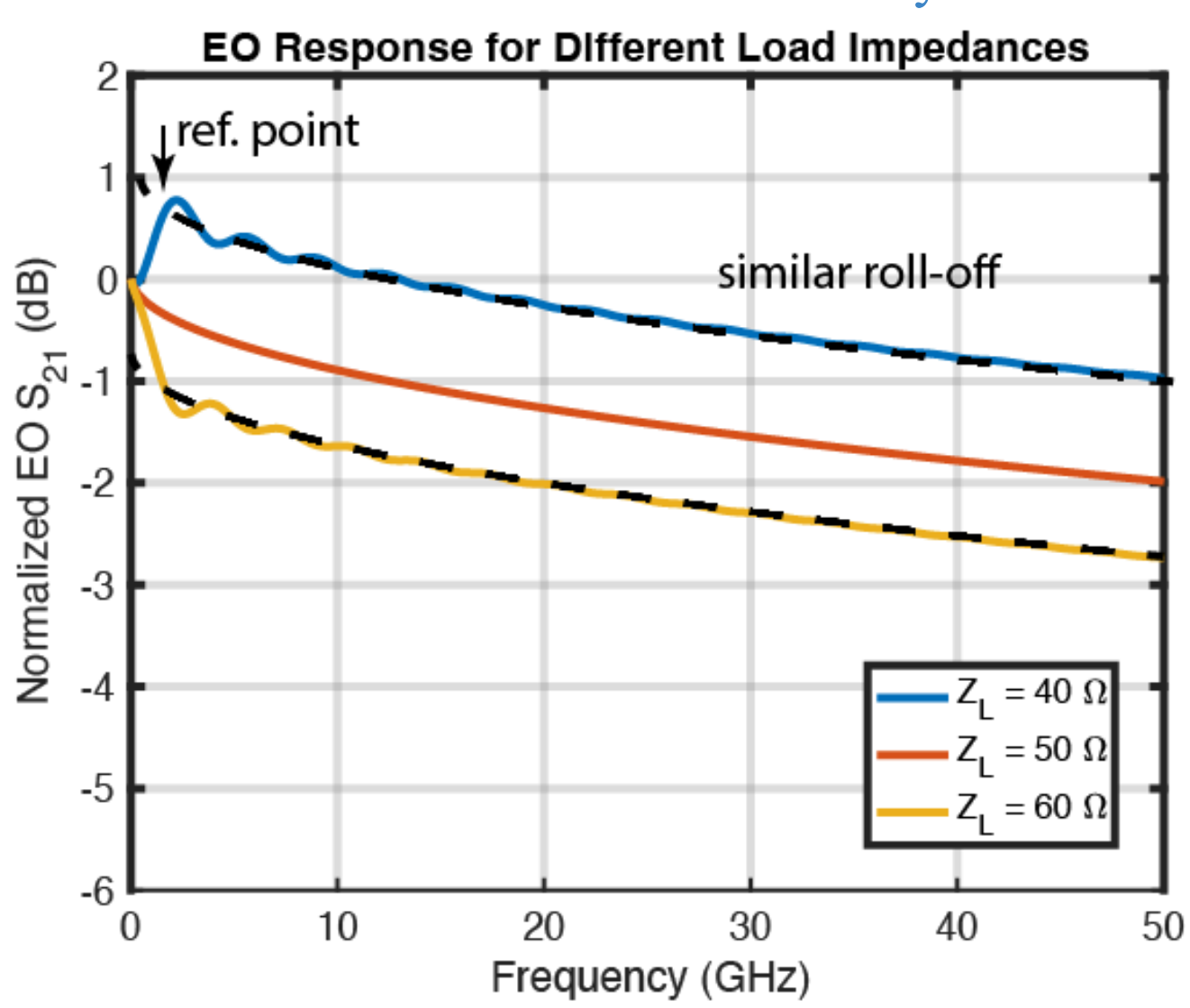

Fig. S3. EO response roll-off fitting under the same condition from Fig. S2a.

This also explains the choice of the reference frequency is somehow not a deterministic value. It all depends on where one can choose that is away from the rise/drop from the DC, thus less affected by the oscillation. It depends on the measurement curve, the length of the modulator, the frequency-dependent Z_0 and Z_L at low frequencies.

It is true that most of the papers (in particular from Loncar Group at Harvard, and its spin off Hyperlight), ref[1-3], who pioneered the TFLN modulator, use 1 GHz as their reference point. In

their recent paper on visible TFLN modulator [Renaud, D., Assumpcao, D.R., Joe, G. et al. Sub-1 Volt and high-bandwidth visible to near-infrared electro-optic modulators. Nat Commun 14, 1496 (2023)], they reference to 3-GHz frequency as shown below [subfigure(c)], which clearly reveals that they pick a reference frequency to avoid the rapid drop off at low frequencies.

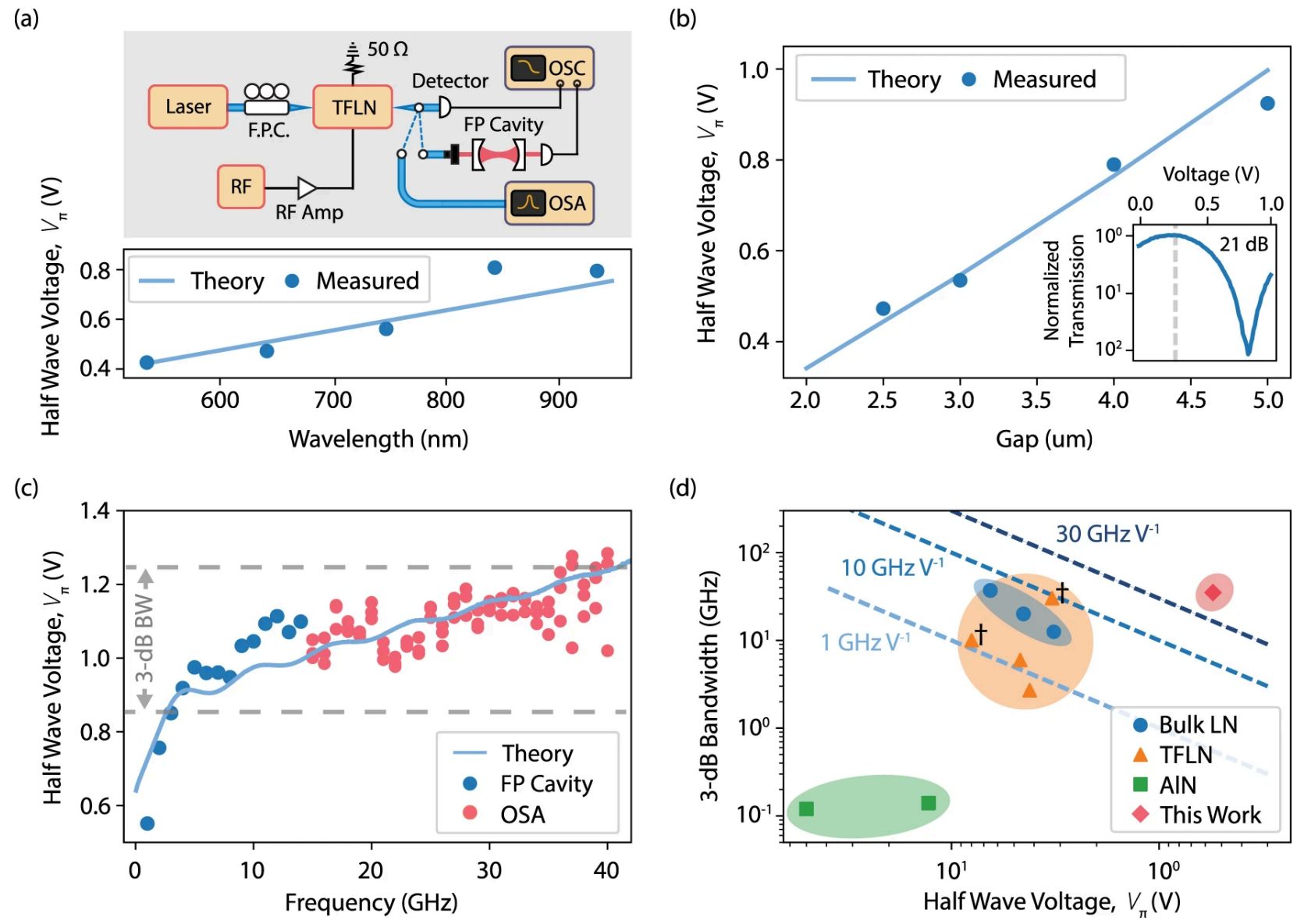

Taken from https://www.nature.com/articles/s41467-023-36870-w [Renaud, D., Assumpcao, D.R., Joe, G. *et al.* Sub-1 Volt and high-bandwidth visible to near-infrared electro-optic modulators. *Nat Commun* **14**, 1496 (2023).] "Fig. 2: Ultra-low Vπ visible-to-near-infrared wavelength Mach-Zehnder modulators with greater than 35 GHz bandwidth. ….**c** Frequency dependence of V_π for a 1 cm long MZM. A 3 dB EO bandwidth of ~ 35 GHz is extracted from the response. The bandwidth is measured with respect to a low frequency reference, here taken to be 3 GHz. **The grey dashed-lines denote the 3 dB bandwidth w.r.t 3 GHz…"**

Moreover, there are demonstrations recently showing that to leverage this initial rise in the EO response by intentionally choosing a lower termination load (ZL less than 50ohm) to demonstrate a higher 3-dB EO bandwidth when referenced to the DC frequency. We list an example below [From Li, Q., Yi, Q., Sun, A. *et al.* Ultra-broadband near- to mid-infrared electro-optic modulator on thin-film lithium niobate. *Nat Commun* **17**, 1138 (2026)]

https://www.nature.com/articles/s41467-025-67902-2
Fig. 3: Measured performance of the thin-film lithium niobate (TFLN) modulator.

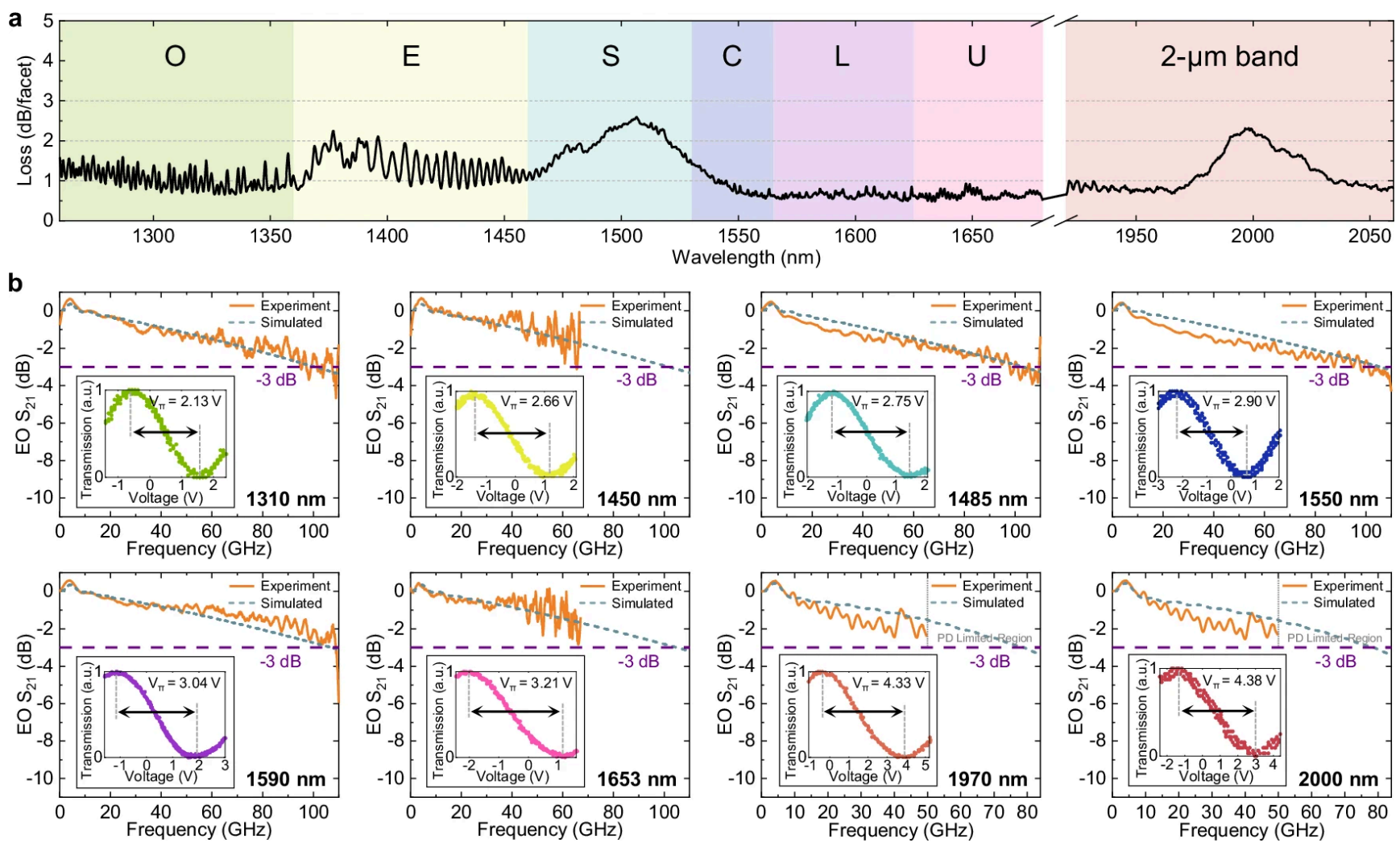

[b] In this figure caption reading "The low-frequency peaks observed in both the simulated and measured responses stem from **the deliberate impedance configuration ($Z_L < Z_0$), which was implemented to increase bandwidth by compensating the drop-off at low frequencies**[37]." Also in the main texts of this paper reading "A load resistor of $Z_L = 38\,\Omega$ is used, which is intentionally lower than Z_0 to mitigate the low-frequency EO S21 roll-off, thereby slightly enhancing the operational bandwidth[37]. .."

Other related work on this phenomenon: "Liu, X. et al. Capacitively-loaded thin-film lithium niobate modulator with ultra-flat frequency response. *IEEE Photonics Technol. Lett.* **34**, 854–857 (2022).; and reported in a much earlier reference in 1987: S. H. Lin and Shih-Yuan Wang, "High-throughput GaAs PIN electrooptic modulator with a 3-dB bandwidth of 9.6 GHz at 1.3 μm," Appl. Opt. 26, 1696-1700 (1987)**.**

In the newly updated ref [Didier, P., Jain, P., Bertrand, M. *et al.* Thin film lithium niobate on sapphire for integrated mid-infrared modulator. *Nat Commun* 17, 3050 (2026).], as also reviewer mentioned as [7]. We think that they also have impedance mismatch (Z0 > Zload regime) based on the fact that the reported EO response goes higher than 0 dB (an initial rise from DC). This is consistent with what they claimed, though not providing the exact value.

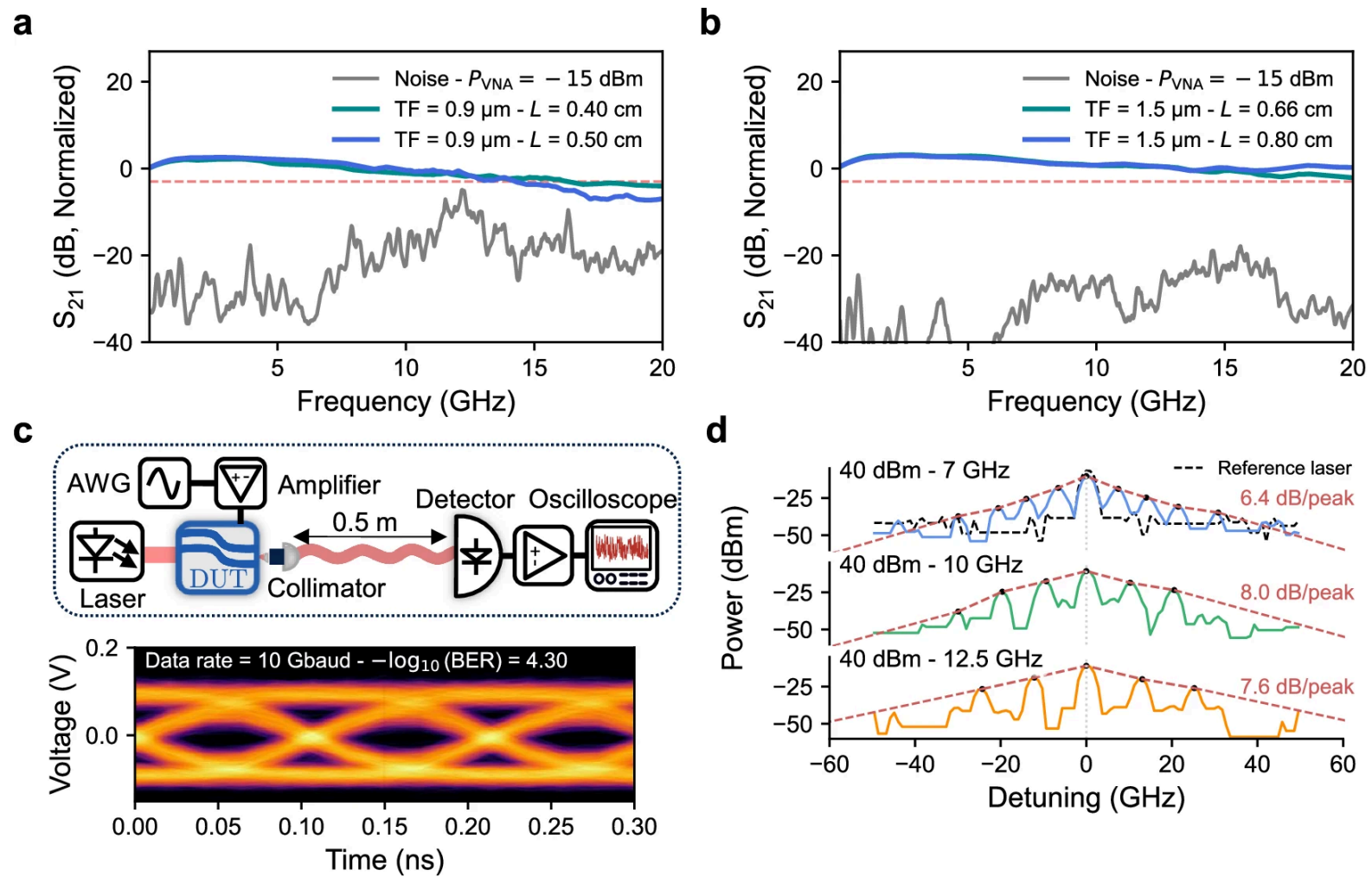

[Didier, P., Jain, P., Bertrand, M. *et al.* Thin film lithium niobate on sapphire for integrated mid-infrared modulator. *Nat Commun* 17, 3050 (2026).], **"Fig. 4: High-speed electro-optic characterization of the MIR MZM.**The data has been normalized by the detector and amplifier responses to most accurately represent the intrinsic electro-optic bandwidth of the modulators. The restricted bandwidth can be attributed to 50 *Ω* impedance mismatch."

Similar behavior can be seen when Z0 is off 50ohm, Zload = 50ohm as well since the reflection can also be positive or negative. The same analysis applies. The more important contribution of Z0 can be extracted from S11, power reflection back to the source which goes with $((Z0-50)/(Z0+50))^2$, which in our case remains lower than -30 dB above 3 GHz (seen in Fig.3b).

Finally, we believe the revised manuscript now clearly explains the rationale for choosing the reference frequency rather than using DC or 1 MHz. In the updated manuscript, we have added new low-frequency Vπ measurements in the revised Fig. 3d, spanning from 100 kHz to 0.4 GHz, where 0.4 GHz is the highest frequency accessible with our mid-infrared photodetector.

As shown in the new data, we observe a rapid roll-off at low frequencies, consistent with the impedance mismatch expected from the measured S11 response between DC and 3 GHz. Because our current measurement setup cannot capture the complete low-frequency roll-off and oscillatory behavior beyond 0.4 GHz, we have revised the reference frequency throughout the manuscript from the previously used 2 GHz (estimated from microwave simulations) to 0.4 GHz, corresponding to the highest experimentally measured frequency before or in the middle of the onset of the low-frequency roll-off.

This revision also enables a more appropriate comparison with most of the prior TFLN electro-optic modulators operating in the telecom and visible wavelength regimes, where Vπ is referenced at frequencies between 1 and 3 GHz rather than at DC. As discussed in the revised manuscript, this convention has been widely adopted because the low-frequency response is often influenced by impedance mismatch, whereas the higher-frequency reference more

faithfully reflects the intrinsic microwave performance of the traveling-wave modulator. We have indicated the new reference point in Fig. 3d together with the measured low-frequency roll-off, allowing readers to directly interpret the complete frequency-dependent response.

Using this updated reference, we extract a 3-dB EO bandwidth of 52 GHz, which is close to our previous estimate of 50 GHz. This value is further supported by an independent consistency check. As a rule of thumb, for an ideal traveling-wave modulator with both microwave-optical index matching and impedance matching, the 3-dB EO bandwidth is expected to coincide with the frequency at which the electrode transmission (S_{21}) reaches approximately −6.4 dB. Our device is index matched, and therefore, when an appropriate reference frequency is chosen, the measured EO bandwidth should closely approach this ideal prediction. From Fig. 3a, the measured electrode transmission is −5.5 dB at 40 GHz (2.75 dB/cm loss for a 2-cm device), corresponding to an estimated −6.4 dB S_{21} frequency of approximately 58 GHz. Since this intrinsic estimate is higher than our experimentally extracted EO bandwidth of 52 GHz, our reported bandwidth should be regarded as reasonable and conservative, representing a lower bound on the intrinsic device performance. The close agreement between these two independent estimates further increases our confidence in the extracted EO bandwidth.

Finally, we would like to emphasize that the absolute frequency-dependent $V\pi$ is ultimately the most meaningful metric for evaluating modulation efficiency and RF power consumption. Therefore, we believe comparisons should be based on the complete $V\pi$ characteristics rather than solely on the normalized electro-optic response or the choice of a single reference frequency. To facilitate such comparisons, we now provide the full low-frequency dataset in the revised manuscript. To the best of our knowledge, our device remains the only demonstrated mid-infrared traveling-wave TFLN modulator achieving an absolute microwave $V\pi$ of 4.5–6.5 V over the 27–34 GHz frequency range through device engineering and high electro-optic efficiency (see Table 1).

We have updated our Figure 3 to be,

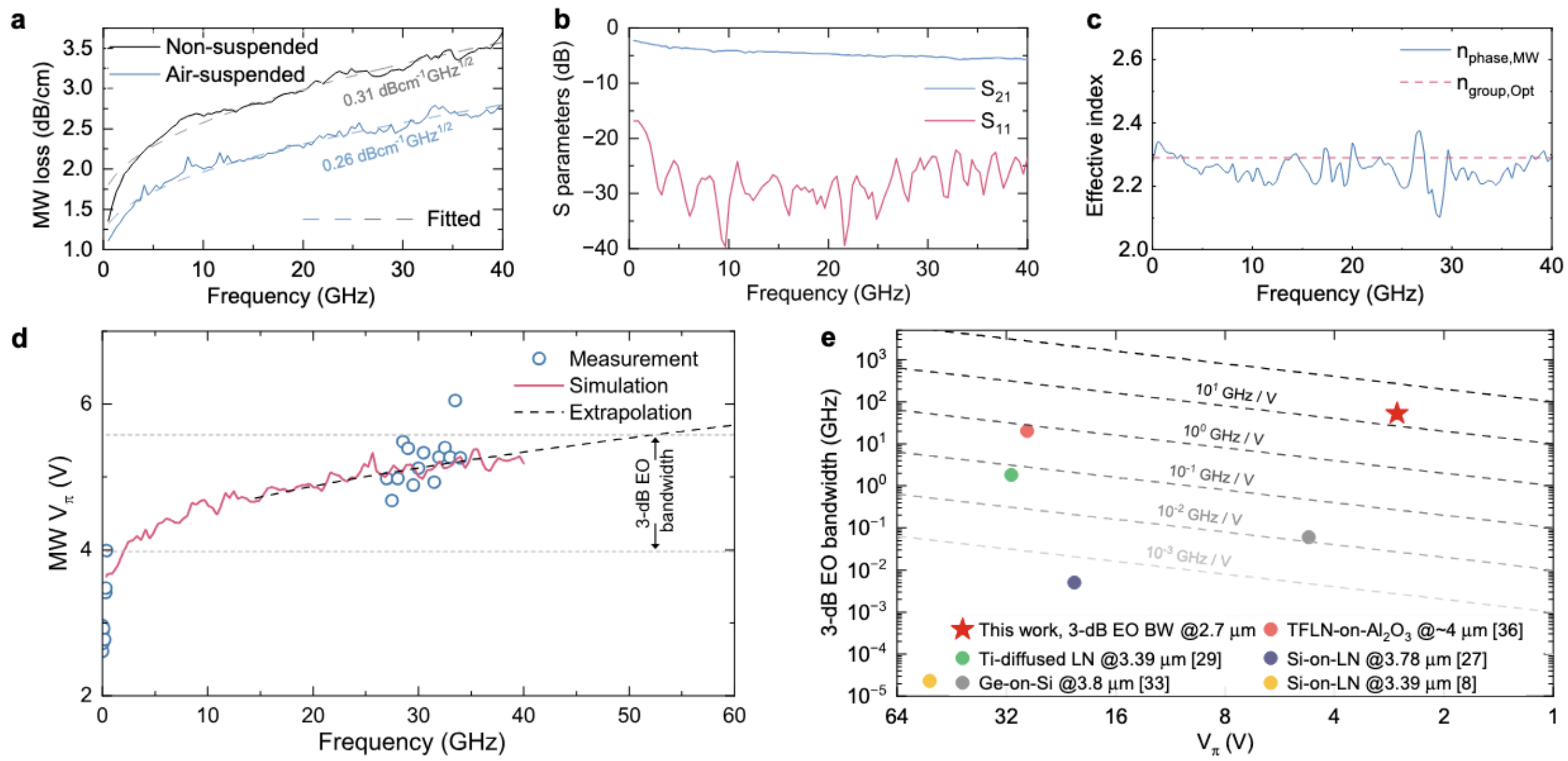

Figure 3: **Characterization of the MIR modulator up to 40-GHz microwave frequencies.** (a) Measured microwave loss on the segmented co-planar electrodes before and after air suspension. Segmented travel-wave electrodes combined with air suspension lead to a reduced MW propagation loss of 2.75 dB/cm at 40 GHz and an ohmic-loss-limited slope of 0.26 dB/cm/$GHz^{0.5}$, which is comparable to the loss slope of the best NIR LN modulators reported [43]. (b) Measured electrical transmission S_{21} and reflection S_{11} spectrum. The measured reflection is below 25 dB from 2 - 40 GHz range indicating a well-matched impedance to 50 Ω. (c) Measured MW phase index, which matches well with the optical group index based on the air-suspended TFLN waveguide (dashed line). (d) Microwave V_π measured from 100 kHz to 34 GHz. Microwave V_π values are measured to be 5 V at 27 GHz and 5.26 V at 34 GHz for a 2-cm-long EO AM at the optical wavelength of 2.7 µm. The microwave V_π is extracted via the optical spectrum from the AM under MW driving. The measurement results match with the simulation based on the measured S parameters in (a). A rapid low-frequency roll-off is observed due to impedance mismatch. Therefore, the EO response is referenced to the measured response at 0.4 GHz, yielding an extracted 3-dB EO bandwidth of 52 GHz (dashed line). The electro-optic response scales inversely with V_π^2 and decreases by only 2.3 dB at 40 GHz, the frequency limit of our signal generator. (e) Comparison of the modulator figure of merit, defined as the ratio between the 3-dB EO bandwidth (BW) and the near-DC V_π. The AM in our work demonstrates significantly higher BW/V_π values of 19.3 GHz/V, as compared to the literature values (the dashed lines indicate constant BW/V_π values).

We have modified the main texts to read,

"...Figure 3d plots the measured microwave half-wave voltage (Vπ) of our 2-cm-long amplitude modulator, which is 4.5–6.5 V over the 27–34 GHz frequency range and agrees well with our simulation based on the device parameters. The Vπ from 100 kHz to 0.4 GHz is measured by the mid-infrared photodetector and also plotted. The direct Vπ measurement between 0.4 GHz and 27 GHz is limited by the available RF amplifier frequency bands and the 7.5-GHz spectral resolution of the Fourier-transform optical spectrum analyzer (Thorlabs OSA205C), since the generated optical sidebands at these frequencies become insufficiently separated to be reliably resolved by the spectrometer. The EO response can be simulated from the measured microwave properties and scales inversely with the square of the microwave Vπ, which matches well with the experimental data (Fig. 3d). It is referenced to a measured non-zero frequency of 0.4 GHz, corresponding to the highest frequency that can be reliably measured with our mid-infrared photodetector before the onset of the impedance-mismatch-induced low-frequency roll-off

[40-42], while still capturing the intrinsic traveling-wave microwave response [42-44]. The EO response decreases by 2.3 dB at a microwave frequency of 40 GHz, which is the frequency limit of our signal generator. By fitting the measured response and referencing the low-frequency (0.4 GHz) response level, the extracted 3-dB EO bandwidth is estimated to be 52 GHz (dashed line). To the best of our knowledge, this represents...
”

2. In Fig. 3d, the authors only measured the performance within 27~34 GHz. Can the performance below 27 GHz also be measured? Adding several data points between the reference frequency and 27 GHz is highly recommended.

We thank the reviewer for this helpful suggestion. The modulator is fully capable of operating below 27 GHz; however, the directly measured microwave Vπ range in the original manuscript was limited by our measurement instrumentation rather than the device itself. Specifically, the high-frequency RF amplifier used for the Vπ characterization operates above approximately 26.5 GHz. Although a lower-frequency amplifier is also available, measurements below 27 GHz produce modulation sidebands that become too closely spaced to be reliably resolved by our Fourier-transform optical spectrum analyzer (Thorlabs OSA205C), which has a spectral resolution of 7.5 GHz. In practice, accurate extraction of Vπ requires the modulation sidebands to be separated by approximately 2–3 times the instrument resolution so that both the carrier depletion and first-order sideband powers can be measured with sufficient accuracy.

To address the reviewer's concern, we have performed additional low-frequency measurements using a calibrated photodetector and updated Fig. 3d to include the measured Vπ from 100 kHz to 0.4 GHz, thereby bridging the gap between DC and our directly measured high-frequency data. These additional measurements provide the missing low-frequency reference while also revealing the expected impedance-mismatch-induced roll-off in the EO response.

We updated our main texts to read,
“...device parameters. The Vπ from 100 kHz to 0.4 GHz is measured by the mid-infrared photodetector and also plotted. The direct Vπ measurement between 0.4 GHz and 27 GHz is limited by the available RF amplifier frequency bands and the 7.5-GHz spectral resolution of the Fourier-transform optical spectrum analyzer (Thorlabs OSA205C), since the generated optical sidebands at these frequencies become insufficiently separated to be reliably resolved by the spectrometer. The EO response..."

3. Ref. 36 has been published as:

Didier, P., Jain, P., Bertrand, M. et al. Thin film lithium niobate on sapphire for integrated mid-infrared modulator. Nat Commun 17, 3050 (2026). https://doi.org/10.1038/s41467-026-69880-5
The reference should be updated.

We thank the reviewer for pointing this out. We have updated Ref. 36 to cite the published version of the article in Nature Communications.

Reply to Reviewer #5

Reviewer #5 (Remarks to the Author):

I co-reviewed this manuscript with one of the reviewers who provided the listed reports. This is part of the Nature Communications initiative to facilitate training in peer review and to provide appropriate recognition for Early Career Researchers who co-review manuscripts.

We thank the co-reviewer for their time and contribution to the evaluation of our manuscript.

Manuscript ID: NCOMMS-25-102205B

"Suspended thin-film lithium niobate modulator for broadband mid-infrared light modulation and frequency comb generation"

Reply to Reviewers' Comments

We thank the Editor and the Reviewers for their careful evaluation of our manuscript. We have addressed the remaining comment and corrected the corresponding error in the revised manuscript, highlighted in red.

The Reviewers' comments are reproduced below in black, followed by our responses in blue.

Reply to Reviewer #4

Reviewer 4 (Remarks to the Author):

The authors have revised the manuscript to address my concerns. However, the revised manuscript still have some errors. For example, Figure 3(b) caption: "The measured reflection is below 25 dB from 2 – 40 GHz range indicating a well-matched impedance to 50 ." It should be -25 dB rather than "25 dB". Moreover, the S11 curve in Fig. 3(b) clearly contains regions above -25 dB, particularly in the 30-40 GHz range. The claim of "below -25 dB from 2 – 40 GHz range" is incorrect. The authors should carefully check their manuscript to avoid such errors.

We thank Reviewer #4 for carefully identifying these errors. We agree that "25 dB" should be corrected to "−25 dB." We also acknowledge that the measured S_{11} exceeds −25 dB over portions of the 30–40 GHz range, and therefore the original statement was inaccurate. We have revised the caption of Fig. 3(b) to accurately describe the measured reflection response and have carefully checked the manuscript for similar errors to read:

"**b**, Measured electrical transmission S_{21} and reflection S_{11} spectrum. The measured reflection is below -20 dB from 2 - 40 GHz range indicating a well-matched impedance to 50 Ω. "

(Page 13)

We sincerely appreciate the reviewer's careful attention, which has helped us improve the accuracy and clarity of the manuscript.

Reviewer 5 (Remarks to the Author):

I co-reviewed this manuscript with one of the reviewers who provided the listed reports. This is part of the Nature Communications initiative to facilitate training in peer review and to provide appropriate recognition for Early Career Researchers who co-review manuscripts.

We thank Reviewer #5 for participating in the co-review of our manuscript and for the careful assessment and constructive feedback.

Key results

The authors demonstrate a suspended thin-film lithium-niobate platform enabling broadband MIR electro-optic modulation with low drive voltages (Vπ = 2.3–4.3 V) across 2.4–3.6 μm wavelengths. They achieve a 40-GHz EO bandwidth) and report high-frequency Vπ ,MW of 4.5–6.5 V up to 35 GHz, representing substantial advances over prior MIR modulators. The work further showcases frequency-agile MIR EO comb generation with 0.8-THz 10-dB optical bandwidth and validates system-level performance in a free-space MIR communication link.

Validity

The manuscript presents strong results and is, in my opinion, suitable for publication after minor revisions. The authors demonstrate noteworthy progress in MIR EO modulation. However, some points require clarification or correction to ensure accuracy and consistency.

1) **Comment on the drawbacks of a suspended structure**

TFLN is known to be sensitive to moisture and environmental factors, therefore usually it is cladded in real world applications. However, the fact that the structure is suspended, cladding it with oxide would not really be possible. It would be beneficial if the authors could comment on potential reliability issues such devices could face if this approach would be scaled up in the future or address approaches how this issue could be solved without hindering the performance metric benefits of such devices.

2) **Clarification of Vπ versus Vπ·L values**

In several parts of the manuscript, the distinction between Vπ and VπL is not consistently maintained, and in some cases the two are used interchangeably. This may create confusion for readers. For example, in the section:

"The corresponding half-wave voltage length product (Vπ·L) are 4.6 V·cm at 2.4 μm and 8.6 V·cm at 3.6 μm wavelength, respectively. The Vπ scales near linearly with the optical wavelength λ … As the suspended optical waveguide supports guided mode over an ultrabroad bandwidth, we tested the Vπ at the NIR wavelength of 1.55 μm to be 1.8 V…."

To improve clarity and enable a direct comparison, I recommend that the VπL value at 1.55 μm be explicitly stated, just as is done for the MIR measurements.

Additionally, the manuscript uses VπL as a figure of merit without clearly specifying that the reported values correspond to a push–pull amplitude modulator, where the effective interaction length is twice the physical arm length. The conventional definition of VπL originates from phase modulators with a single-arm interaction length. If the authors indeed report the effective push–pull VπL, this should be clearly stated to avoid ambiguity and ensure correct interpretation.

3) Incorrect referencing in Figures 2c and 3e

In both Figure 2c and Figure 3e, the numbering of the references in the plot does not match the references in Table 1 and the Bibliography. This must be corrected to avoid confusion and ensure that the comparisons presented in the figures can be accurately traced back to their corresponding sources. I recommend carefully rechecking all figure reference indices in the text and Table 1.

4) Use of BW/ Vπ (GHz/V) as a figure of merit

The manuscript uses BW/Vπ (GHz/V) as a figure of merit and compares it with prior modulators. This FOM is only valid in an oversimplified scenario as its validity relies on the assumption that the EO bandwidth scales inversely with interaction length under attenuation-limited, near velocity-matched conditions. Standard analytical formulations in the literature explicitly show that in general, linear length dependence for bandwidth cannot be directly assumed [1], [2], [3].
Since different works may be limited by different mechanisms (like RF attenuation, velocity mismatch, parasitics etc), cross-paper comparison using BW/Vπ may not be physically equivalent, thus being a somewhat meaningless way of comparison.

I recommend that the authors either

(i) clarify the physical regimes under which this FOM is meaningful and explicitly state the assumptions behind the comparison with other works, or

(ii) consider omitting this FOM from the text and figures as the manuscript already demonstrates superior performance relative to the literature.

[1] D. Zhu *et al.*, “Integrated photonics on thin-film lithium niobate,” *Advances in Optics and Photonics*, vol. 13, no. 2, p. 242, May 2021, doi: https://doi.org/10.1364/aop.411024

[2] J. Witzens, “High-Speed Silicon Photonics Modulators,” *Proceedings of the IEEE*, vol. 106, no. 12, pp. 2158–2182, Dec. 2018, doi: https://doi.org/10.1109/jproc.2018.2877636

[3] G. Ghione, “Semiconductor Devices for High-Speed Optoelectronics,” Oct. 2009, doi: https://doi.org/10.1017/cbo9780511635595
